# Uncovering the Key Role of Distortion in Bioorthogonal Tetrazine Tools That Defy the Reactivity/Stability Trade-Off

**DOI:** 10.1021/jacs.2c01056

**Published:** 2022-05-02

**Authors:** Dennis Svatunek, Martin Wilkovitsch, Lea Hartmann, K. N. Houk, Hannes Mikula

**Affiliations:** † Institute of Applied Synthetic Chemistry, TU Wien 1060 Vienna, Austria; ‡ Department of Chemistry and Biochemistry, University of California, Los Angeles, Los Angeles 90095, United States

## Abstract

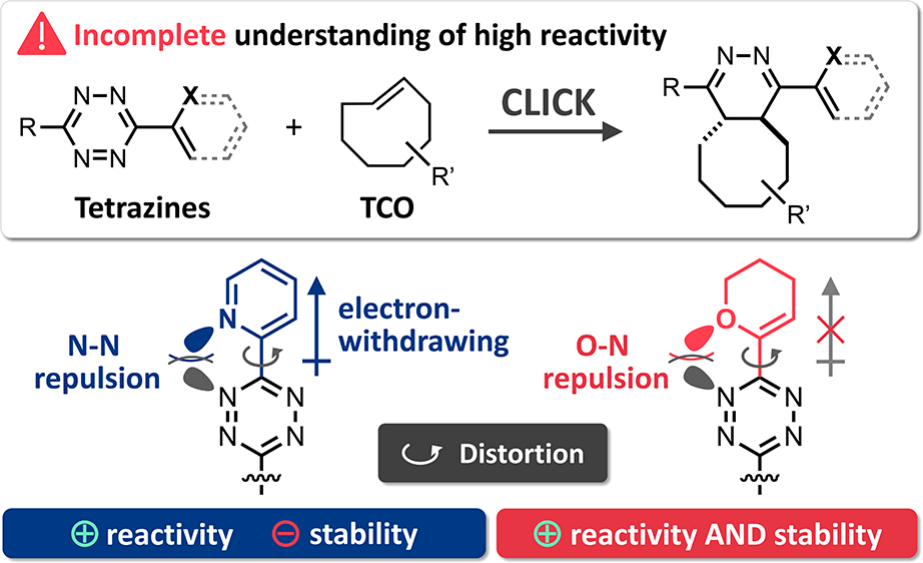

The tetrazine/*trans*-cyclooctene ligation stands out from the bioorthogonal toolbox due to its exceptional reaction kinetics, enabling multiple molecular technologies in vitro and in living systems. Highly reactive 2-pyridyl-substituted tetrazines have become state of the art for time-critical processes and selective reactions at very low concentrations. It is widely accepted that the enhanced reactivity of these chemical tools is attributed to the electron-withdrawing effect of the heteroaryl substituent. In contrast, we show that the observed reaction rates are way too high to be explained on this basis. Computational investigation of this phenomenon revealed that distortion of the tetrazine caused by intramolecular repulsive N–N interaction plays a key role in accelerating the cycloaddition step. We show that the limited stability of tetrazines in biological media strongly correlates with the electron-withdrawing effect of the substituent, while intramolecular repulsion increases the reactivity without reducing the stability. These fundamental insights reveal thus far overlooked mechanistic aspects that govern the reactivity/stability trade-off for tetrazines in physiologically relevant environments, thereby providing a new strategy that may facilitate the rational design of these bioorthogonal tools.

## Introduction

**Figure 1:**
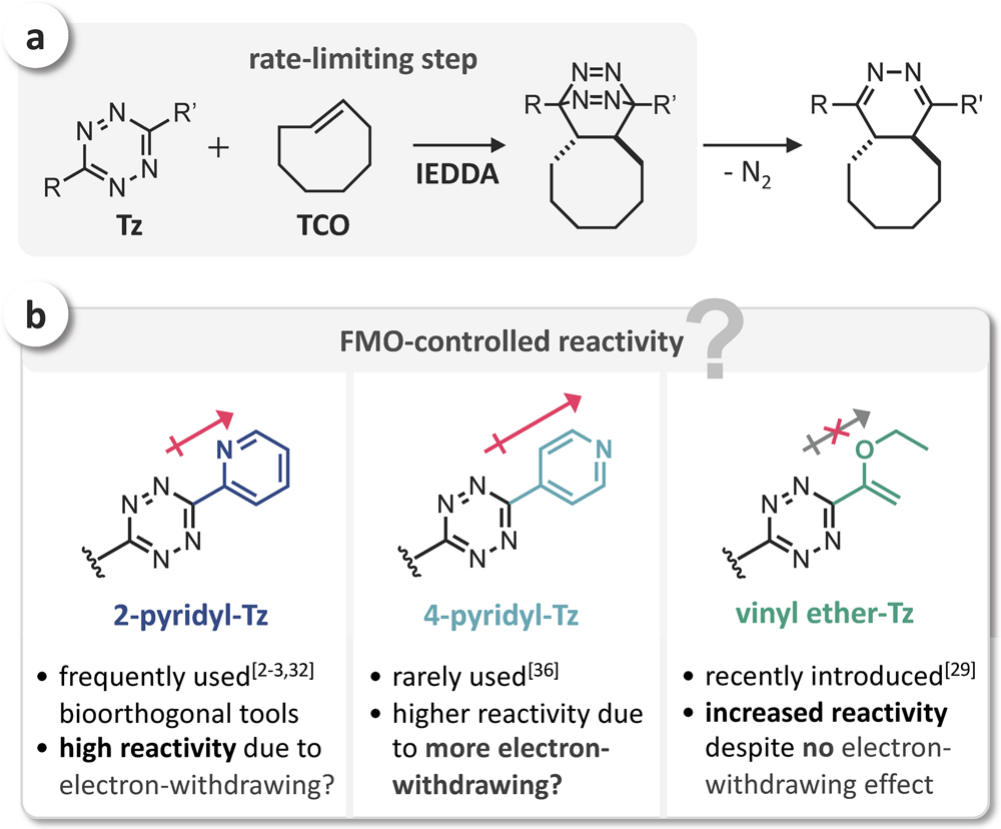
(a) IEDDA-initiated Tz ligation with **TCO**; (b) 2-pyridyl-,^2,3,32^ 4-pyridyl-,^36^ and recently introduced^29^ vinyl ether-Tz.

The inverse electron demand Diels–Alder (IEDDA)-initiated ligation of 1,2,4,5-tetrazines (Tz) with strained dienophiles represents a group of exceptionally fast bioorthogonal reactions.^1−3^ In particular, *trans*–cyclooctene (**TCO**) derivatives^4−6^ provide several orders of magnitude higher reactivity than other dienophiles such as cyclopropenes^7^ or norbornenes.^8^ In the rate-determining step, the tetrazine first reacts with the *trans*-cyclooctene in a [4 + 2]-cycloaddition reaction to form a bicyclic intermediate that rapidly undergoes cycloreversion to give dihydropyridazines as ligation products (Figure 1a). Due to high reaction rates, its biocompatibility, and versatility, the tetrazine/*trans*-cyclooctene ligation has found broad applications in many fields, in particular enabling selective chemical reactions in living organisms.^2,3^ Very recently, bioorthogonal chemistry has entered phase 1 clinical trials, with tetrazine/*trans*-cyclooctene reactions currently being tested in humans, aiming for locally restricted prodrug activation to improve the selectivity of chemotherapeutics.^9,10^

In recent years, a variety of differently substituted Tz have been used for bioorthogonal reactions and in vivo chemistry, including bis-alkyl-substituted Tz,^11−15^ alkyl-aryl-Tz,^16−19^ mono-alkyl-Tz^20^ (alkyl-H-Tz) as well as highly reactive bis-heteroaryl^21,22^ and mono-aryl^23−26^ derivatives (aryl-H-Tz). These applications have motivated and fueled the development of advanced procedures for the synthesis of Tz scaffolds.^27−31^ In particular, 2-pyridyl-Tz are frequently used despite limited stability because of their exceptionally high reactivity. This is commonly attributed to the electron-withdrawing effect of the 2-pyridyl substituent, resulting in a lowered orbital energy of the Tz, thereby accelerating the IEDDA cycloaddition (Figure 1b).^2,3,32^ Assuming that the reactivity is indeed controlled by frontier molecular orbital (FMO) interactions, we hypothesized that 4-pyridyl-substituted Tz are even more reactive than their 2-pyridyl analogues.^33−35^ So far, 4-pyridyl-Tz (Figure 1b) have only rarely been used,^36^ and there is no comparative data on the IEDDA reaction kinetics of 2-pyridyl- and 4-pyridyl-Tz. In addition, vinyl ether-Tz, recently introduced by Fox et al.,^29^ have been shown to exhibit increased IEDDA reactivity despite the non-electron-withdrawing nature of the substituent (Figure 1b), while also maintaining high stability. Intrigued by these findings, we speculated about the existence of yet overlooked mechanistic aspects other than FMO interactions that have a crucial effect on the reactivity of Tz.

Here, we present the results of a combined experimental and computational approach, finally revealing the key role of Tz distortion in the bioorthogonal reaction with *trans*-cyclooctenes. We show that this effect accelerates both 2-pyridyl- and vinyl ether-substituted Tz, thereby providing fundamental insight into the underlying mechanism and a molecular basis to describe the reactivity and stability of these bioorthogonal tools.

## Results and Discussion

**Figure 2:**
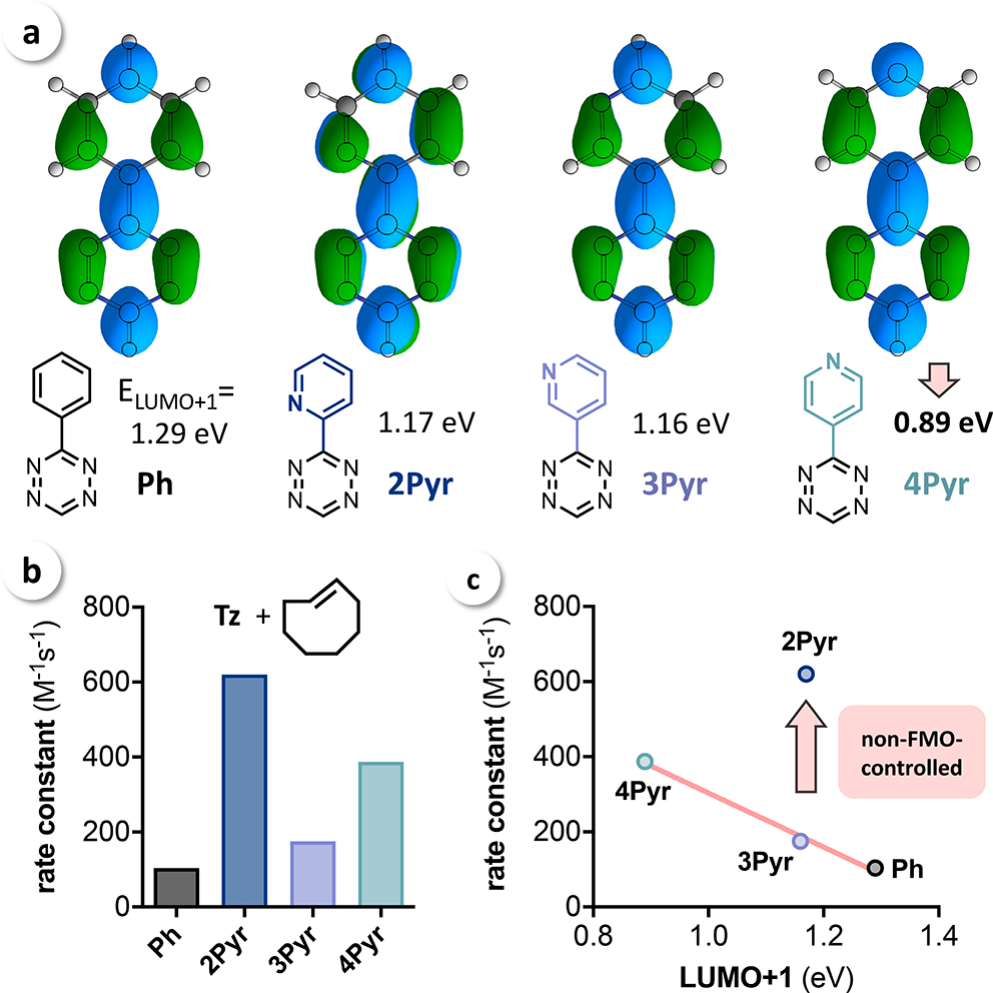
(a) LUMO+1 orbitals and orbital energies of mono-substituted Tz showing a significantly lower LUMO+1 energy for **4Pyr**; (b) rate constants for the reaction of **Ph**, **2Pyr**, **3Pyr**, and **4Pyr** with **TCO** (1,4-dioxane, 25 °C, *n* = 6, SD < 1%); (c) measured rate constants vs calculated LUMO+1 energy.

First, we aimed to study the influence of aryl substituents on the click reactivity with **TCO** and therefore selected a series of phenyl (**Ph**)-, 2-pyridyl (**2Pyr**)-, 3-pyridyl (**3Pyr**)-, and 4-pyridyl (**4Pyr**)-substituted Tz (Figure 2a) for initial investigations. Theoretical calculations were performed using density functional theory (DFT) at the ωB97X-D/6-311G(d,p)-SMD(1,4-dioxane) level of theory, and orbital energies were calculated at the HF/6-311+G(d,p)-SMD(1,4-dioxane)//ωB97X-D/6-311G(d,p)-SMD(1,4-dioxane) level of theory. A detailed description of the computational methods used within this study can be found in the Supporting Information. Energies for the reacting LUMO+1^32^ of Tz range from 1.29 to 0.89 eV. As expected, **Ph** shows the highest orbital energy, followed by **2Pyr** and **3Pyr**, which were calculated to have an almost equal LUMO+1 level, while **4Pyr** shows the lowest orbital energy (Figure 2a). According to FMO theory, **4Pyr** should thus indeed show an increased IEDDA reactivity compared to **2Pyr**.

To compare these results with the measured reaction rates, we have prepared all selected mono-substituted aryl-Tz using a method recently published by Audebert and co-workers.^27^ The rate constants for the ligation with **TCO**(37) were measured by monitoring the IEDDA reactions in 1,4-dioxane at 25 °C by stopped-flow spectrophotometry. The measured rate constants range from 100 M^–1^ s^–1^ for **Ph** to 620 M^–1^ s^–1^ for **2Pyr** (Figure 2b). While the reactivity trend for **Ph**, **3Pyr**, and **4Pyr** seems to be governed by FMO interactions, **2Pyr** is significantly more reactive (>3-fold) than expected based on the respective orbital energy (Figure 2c). Qualitatively equivalent results were obtained using the calculated Kohn–Sham orbital energies (see Supporting Information, Figure S1). Hence, the high IEDDA reactivity of **2Pyr** cannot be attributed to the electron-withdrawing effect of the 2-pyridyl substituent.

**Figure 3:**
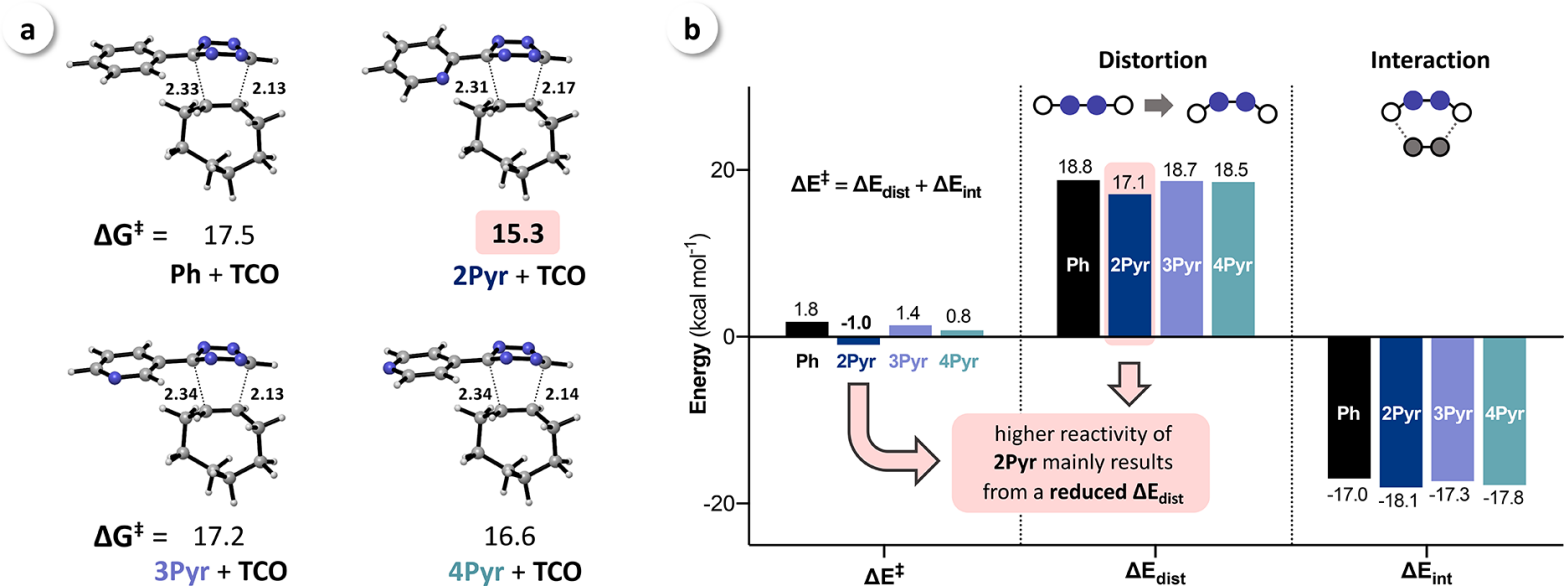
(a) Calculated transition-state geometries and free energies of activation (Δ*G*^⧧^, kcal mol^–1^) for the reaction of mono-substituted Tz (**Ph**, **2Pyr**, **3Pyr**, and **4Pyr**) and **TCO**; (b) distortion/interaction analysis shows that the high reactivity of 2-pyridyl-substituted Tz results from a reduced distortion energy (Δ*E*_dist_).

To investigate the origin of the observed reactivity trend for different pyridyl substituents, a DFT study of the respective reactions was performed, showing good correlation between the calculated free energies of activation (Δ*G*^⧧^) and the experimental values (Figure S2). The calculated transition-state geometries revealed a slight asynchronicity with very similar forming bond lengths across all four Tz. However, **2Pyr** shows a slightly more synchronous bond formation (Figure 3a).

For a detailed investigation of the barrier heights, we performed a distortion/interaction analysis (also referred to as the activation/strain model)^38^ on all four transition states (Figure 3b) using the auto DIAS software package.^39^ This energy decomposition method was introduced independently by Houk and Bickelhaupt and has successfully been applied to investigate bioorthogonal cycloadditions.^32,40−42^ In this analysis, the energy of activation (Δ*E*^⧧^) is dissected into two parts, the distortion energy (Δ*E*_dist_), which is needed to distort the isolated reactants into transition-state geometry, and the interaction energy (Δ*E*_int_) resulting from bringing the two distorted isolated fragments together. The analysis was performed at the transition state as a defined point for each reaction. Performing the distortion/interaction analysis at the transition state geometry can lead to skewed results if transition states at highly different forming bond lengths are compared.^43^ However, here the forming bond lengths are very similar in all cases, warranting the comparison at the transition state. To validate these results, an analysis at a consistent geometry with fixed bond lengths for all Tz was conducted, which qualitatively provided the same results (see Supporting Information, Table S2). Interaction energies were calculated to be similar for all studied reactions (within 1.1 kcal mol^–1^) and do not explain the observed differences in IEDDA reactivity. However, Δ*E*_dist_ for **2Pyr** is about 1.5 kcal mol^–1^ lower than that for **Ph**, **3Pyr**, and **4Pyr,** demonstrating that the increased reactivity of **2Pyr** with **TCO** is mainly caused by a reduced distortion energy (Figure 3b).

**Figure 4:**
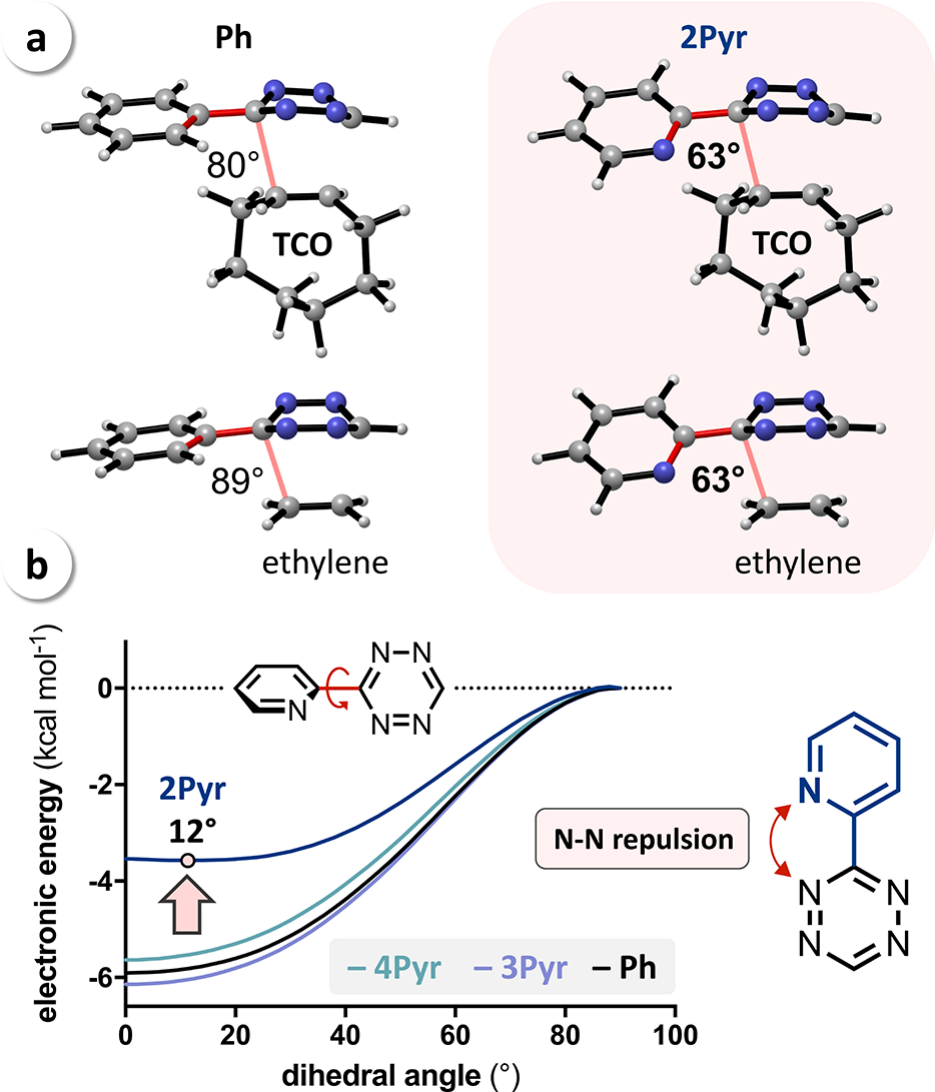
(a) Dihedral angle in the transition states for the reaction of **Ph** and **2Pyr** with **TCO** and ethylene (for **3Pyr** and **4Pyr,** see Figure S3); (b) calculated energy profiles for the rotation of the aryl–Tz bond showing that N–N repulsion reduces the stabilization energy for **2Pyr**.

To explain the lowered Δ*E*_dist_ for aryl substituents with nitrogen atoms in 2-position, we have focused on the geometries encountered at the transition state. In all cases, the aryl moiety is tilted away from the allylic CH_2_ of **TCO**. For **Ph**, **3Pyr**, and **4Pyr,** the dihedral angle is approximately 80°, which we reasoned is due to the steric demand of the allylic CH_2_. This hypothesis is in agreement with the investigations of analogous reactions with ethylene (no allylic CH_2_), showing dihedral angles of approx. 90° (Figures 4a and S3). However, for **2Pyr,** we observed a much stronger tilt in the transition state for the reaction with **TCO**, with a dihedral angle of 63°, which did not change in the reaction with ethylene. These observations demonstrate that it is an intrinsic property of 2-pyridyl-substituted Tz rather than forced by steric interactions. In fact, the calculated geometry of **2Pyr** revealed that a nitrogen–nitrogen interaction destabilizes the reactant. This interaction becomes apparent when looking at the stabilization energies when going from an orthogonal (i.e., dihedral angle between the two aromatic rings of 90°) conformer in the reactant to the stable, coplanar conformer (Figure 4b). For **Ph**, **3Pyr**, and **4Pyr,** this stabilization energy based on the conjugation between the ring systems is approximately 6 kcal mol^–1^. However, for **2Pyr,** the most stable conformation is not planar, and the stabilization is only 3.5 kcal mol^–1^ due to a repulsive interaction between the pyridyl nitrogen and the vicinal Tz nitrogen. This repulsion counteracts the stabilization due to the conjugation of the aromatic systems, leading to an almost flat energy surface between −30 and +30°, with a minimum at a dihedral angle of 12° (Figure 4b). At the transition state, this N–N repulsion can be avoided by the rotation of the substituent, thereby increasing the distance between the interacting nitrogen atoms. The nitrogen lone pairs then point in different directions, further reducing the repulsive interaction. In addition, natural bond orbital analysis revealed (i) a weak intermolecular hydrogen bond between the pyridyl nitrogen of **2Pyr** and the vinylic CH of **TCO**, similar to the interactions previously described for bioorthogonal 1,3-dipolar cycloadditions^44^ and (ii) an increased n_N_ → σ_C–N_* donation of the pyridyl lone pair into the σ* of the Tz C–N bond.^45^ However, both these weak interactions were found to play a minor or negligible role regarding the observed rotation in the transition state (see Supporting Information, Figure S4).

In summary, our results revealed that the high reactivity of 2-pyridyl-Tz cannot be explained by the electron-withdrawing nature of the heteroaryl substituent only but moreover by intramolecular N–N repulsion, finally uncovering the mechanistic key role of distortion in Tz ligations.

**Figure 5:**
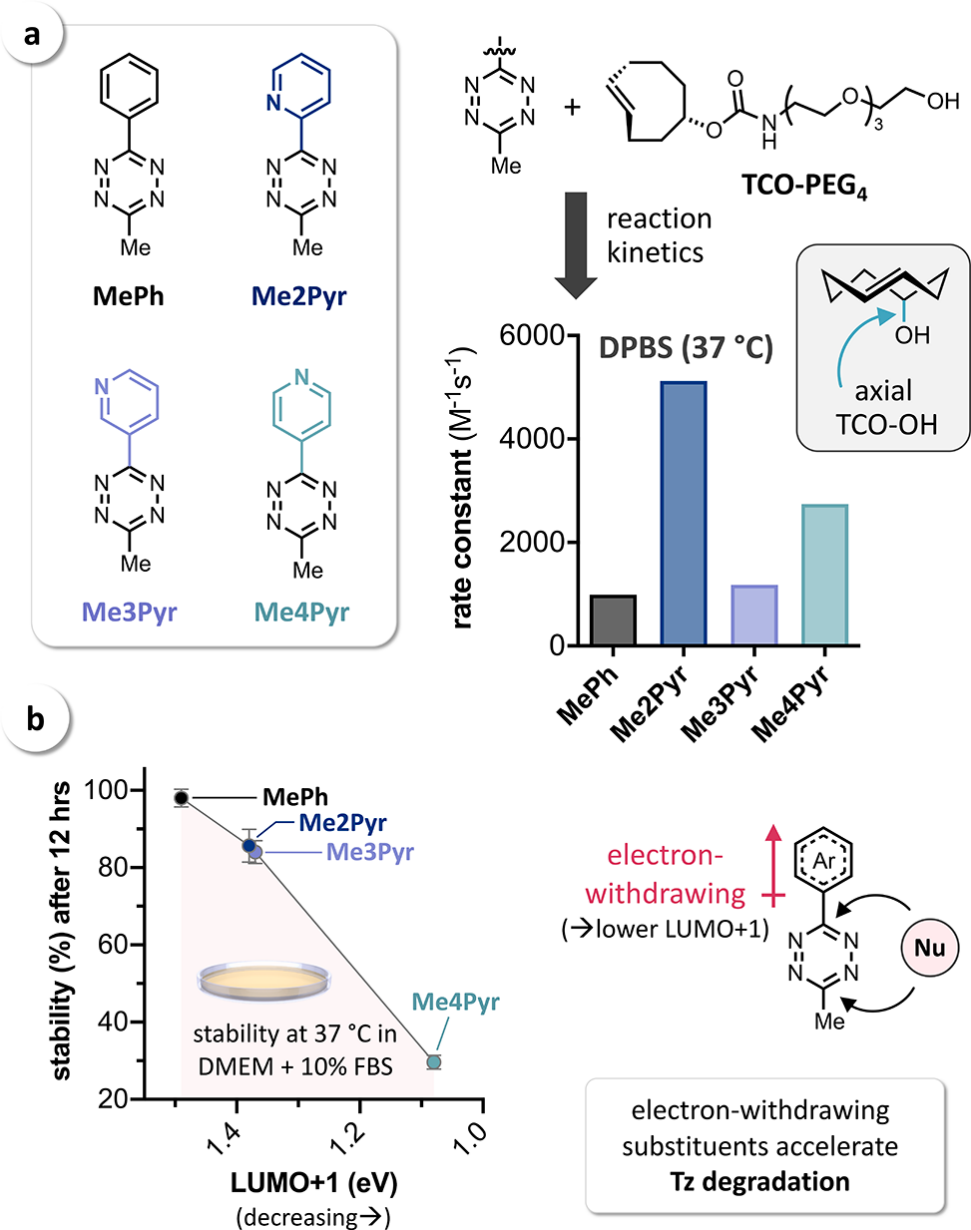
(a) Reaction kinetics of **MePh**, **Me2Pyr**, **Me3Pyr,** and **Me4Pyr** in buffered aqueous solution (DPBS) at 37 °C using water-soluble **TCO-PEG**_**4**_ (*n* = 6, SD < 1%); (b) stability of Tz in cell growth medium at 37 °C (*n* = 3) revealing accelerated degradation with decreasing LUMO+1 energy as a measure for the increased electron-withdrawing character of the (hetero)aryl substituent, promoting the attack of nucleophiles (Nu).

To confirm that these findings can be translated to physiological conditions, additional IEDDA reactions were carried out in Dulbecco’s phosphate buffered saline (DPBS) at 37 °C. Due to the limited stability of the mono-substituted H-Tz in aqueous solution, we have prepared the respective methyl-Tz **MePh**, **Me2Pyr**, **Me3Pyr**, and **Me4Pyr** and determined the second-order rate constants for the reaction with water-soluble **TCO–PEG**_**4**_ (Figure 5a). This **TCO** derivative has been prepared starting from axially configured TCO–OH, a frequently used tag for the design of in vivo chemical tools.^22,46,47^ The observed relative reactivity profile almost exactly matches the data as previously obtained for the reactions of the corresponding aryl-H-Tz with **TCO** in 1,4-dioxane (cf. Figure 2b), with **Me2Pyr** showing the highest rate constant in the reaction with **TCO-PEG**_**4**_ (Figure 5a).

It is generally accepted that increasing the reactivity of Tz by using electron-withdrawing substituents leads to reduced stability in aqueous/biological media due to accelerated attack of nucleophiles causing Tz degradation (Figure 5b).^1−3,48,49^ However, when working with buffered aqueous solutions of the aryl-methyl-Tz, we noticed a significantly faster degradation of **Me4Pyr** compared to **Me2Pyr** (as indicated by accelerated fading of the characteristic pink color of Tz), despite the higher reactivity of the 2-pyridyl-Tz. To investigate this observation, **MePh**, **Me2Pyr**, **Me3Pyr**, and **Me4Pyr** were incubated in full cell growth medium (Dulbecco’s modified Eagle’s medium, DMEM, incl. 10% fetal bovine serum) at 37 °C, and Tz stability was monitored by spectrophotometry (Figure 5b). Indeed, **Me4Pyr** degraded much faster (30% intact after 12 h) compared to **Me3Pyr** and **Me2Pyr** (approx. 85%) and **MePh** (>95%). These results correlate well with the decreasing LUMO+1 energy^32^ of the Tz (Figure 5b), which we used as a measure for the increased electron-withdrawing effect of the (hetero)aryl substituent. This finding is in agreement with the hypothesis of accelerated nucleophilic attack leading to Tz degradation. **Me2Pyr** is thus not only more reactive than predicted by FMO theory but also shows a significantly higher stability than expected based on its IEDDA reactivity.

**Figure 6:**
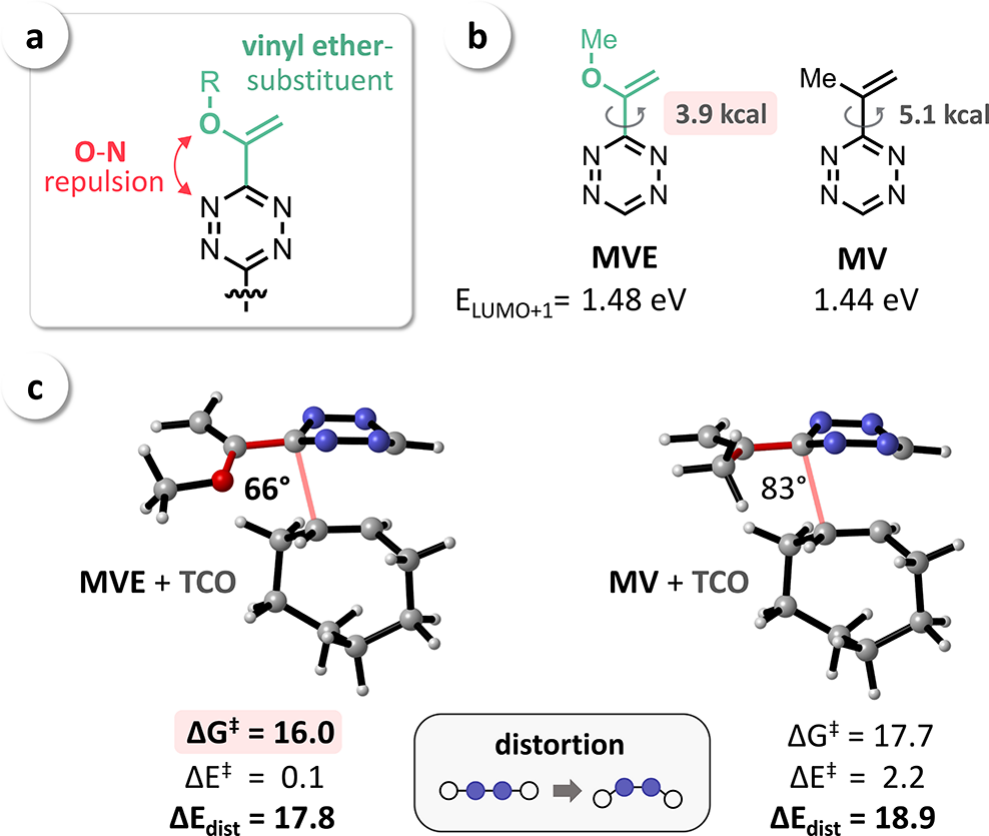
(a) Repulsive O–N interaction increases the reactivity of vinyl ether-Tz; (b) computational analysis revealed a reduced rotational barrier for **MVE** in comparison to **MV** and a relatively high LUMO+1 energy of 1.46 eV; (c) optimized transition-state geometries (Tz + **TCO**) and distortion/interaction analysis confirmed O–N repulsion to be the main reason for the increased reactivity of **MVE**, as indicated by the calculated values for Δ*G*^⧧^, Δ*E*^⧧^, and Δ*E*_dist_ (kcal mol^–1^).

Based on these key mechanistic insights and considering the mentioned vinyl ether-substituted Tz developed by Fox and co-workers (Figure 1b),^29^ we hypothesized that the observed unexpectedly high reactivity of these Tz is due to the lowered distortion energies caused by intramolecular O–N repulsion (Figure 6a), analogous to N–N repulsion in 2-pyridyl-substituted Tz. Considering the non-electron-withdrawing character of the vinyl ether moiety, this would moreover explain the high stability of these Tz despite the increased IEDDA reactivity. To confirm our assumption, we performed computational investigations using the Tz structures **MVE** (methylvinyl ether-Tz) and **MV** (methylvinyl-Tz). The optimized geometries revealed that an oxygen–nitrogen interaction leads to a reduced rotational barrier of 3.9 kcal mol^–1^ for **MVE** (similar to **2Pyr**) in comparison to 5.1 kcal mol^–1^ for **MV** (Figure 6b). The calculated LUMO+1 energies moreover indicate a non-electron-withdrawing character of both substituents (∼1.5 eV in contrast to 0.89 eV, as calculated for **4Pyr**). The transition-state geometries for the reaction with **TCO** (Figure 6c) showed a significantly stronger tilt of the vinyl–Tz bond in the case of the vinyl ether-Tz **MVE** (dihedral angle of 66°). The calculated distortion energies (Δ*E*_dist_) finally confirmed O–N repulsion to play a crucial role regarding the potentially increased reactivity of **MVE**, as indicated by the calculated free energy of activation (Δ*G*^⧧^) of 16.0 kcal mol^–1^.

**Figure 7:**
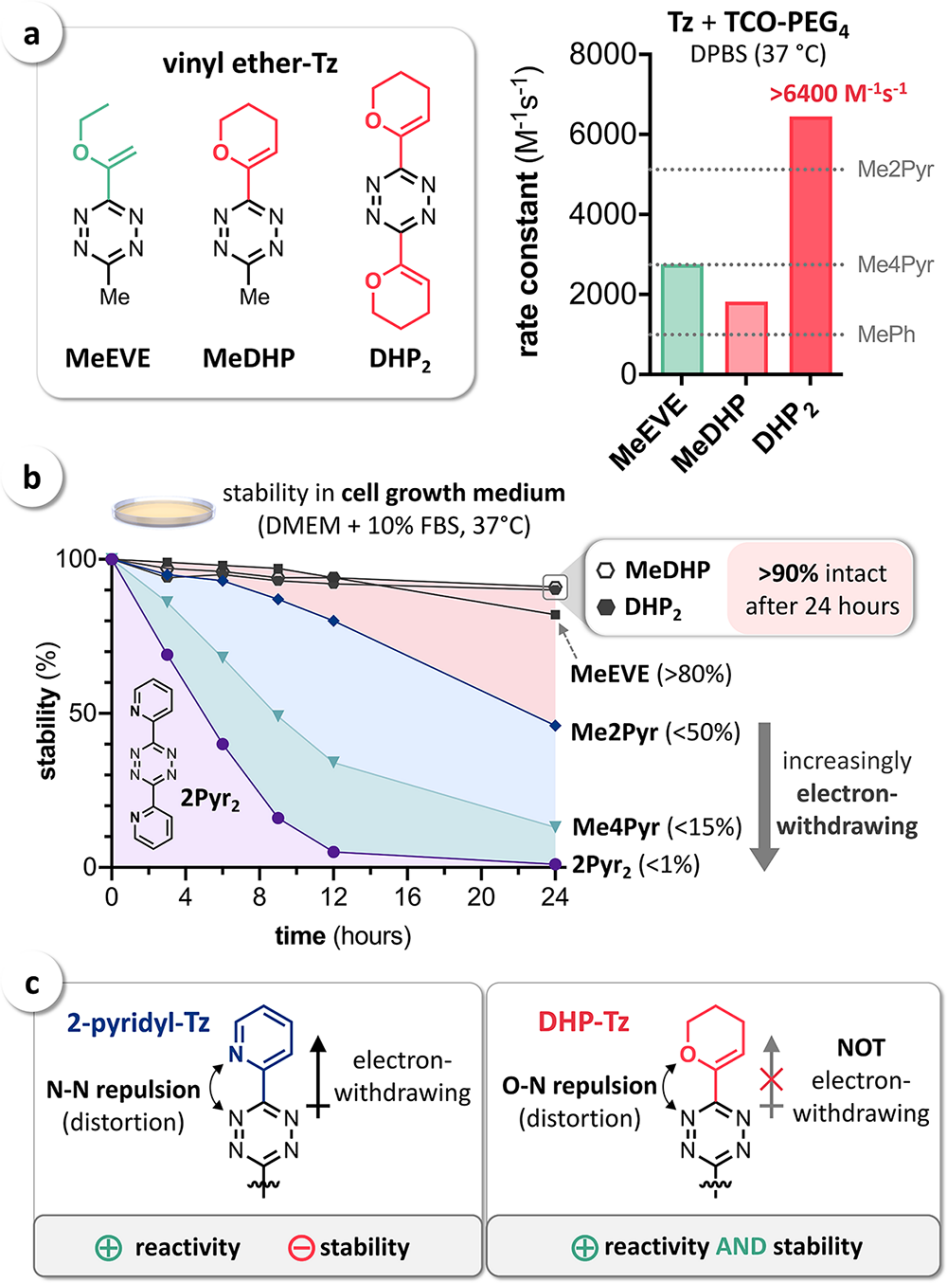
(a) Vinyl ether-Tz **MeEVE**, **MeDHP,** and **DHP**_**2**_ and second-order rate constants for the reaction with **TCO-PEG**_**4**_ in buffered aqueous solution (DPBS) at 37 °C (*n* = 6, SD < 1%); (b) stability of pyridyl- and vinyl ether-Tz under physiological conditions (full cell growth medium, 37 °C, *n* = 3, SD < 5%) revealing accelerated degradation of pyridyl-Tz in contrast to the exceptional stability of DHP-substituted Tz; (c) 2-pyridyl substituents increase the IEDDA reactivity of Tz, also leading to limited stability due to the electron-withdrawing effect. In contrast, non-electron-withdrawing vinyl ether substituents such as DHP increase the reactivity via intramolecular repulsion, without sacrificing stability.

Encouraged by the computational results, we have prepared the vinyl ether-Tz **MeEVE** and the 3,4-dihydro-2*H*-pyran (DHP)-substituted Tz **MeDHP** and **DHP**_**2**_ (Figure 7a; for details on synthetic procedures, see the Supporting Information), but did not obtain sufficient amounts of pure material of the respective bis(vinyl ether)Tz **EVE**_**2**_ [3,6-bis(1-ethoxyvinyl)Tz; structure not shown]. Second-order rate constants for the reactions of these Tz with **TCO-PEG**_**4**_ in DPBS at 37 °C were determined by stopped-flow spectrophotometry. The IEDDA reactivity of **MeEVE** (2750 M^–1^ s^–1^) was shown to match the value measured for **Me4Pyr** (2740 M^–1^ s^–1^). In comparison, the cyclic vinyl ether-Tz **MeDHP** was observed to be less reactive (1820 M^–1^ s^–1^), though still significantly faster than the aryl-Tz **MePh** (990 M^–1^ s^–1^). As expected, we observed a high reactivity of the bis-vinyl ether-Tz **DHP**_**2**_ (6450 M^–1^ s^–1^), exceeding the rate constant of **Me2Pyr** (5120 M^–1^ s^–1^) by approx. 25% (Figure 7a). These results finally confirm the distortion-induced IEDDA acceleration due to intramolecular O–N repulsion and that increased reactivities similar to pyridyl-Tz can be achieved by using non-electron-withdrawing vinyl ether substituents.

Subsequent investigation of Tz stability in full cell growth medium at 37 °C moreover confirmed the high stability of vinyl ether-Tz **MeEVE** (in accordance with previous findings^29^) and, in particular, **MeDHP** and **DHP**_**2**_, in contrast to the limited stability of pyridyl-substituted Tz (Figure 7b). For instance, despite being equal in reactivity, **MeDHP** is significantly more stable than **4Pyr** (>90% vs <15%). Notably, DHP substituents do not lead to decreased stability, as shown by the data obtained for symmetrical **DHP**_**2**_ and bis(2-pyridyl)Tz (**2Pyr**_**2**_). While installation of a second DHP had no detrimental effect on stability (>90% for both **MeDHP** and **DHP**_**2**_), an additional 2-pyridyl substituent resulted in almost complete Tz degradation within 24 h. Despite showing very fast IEDDA reaction with **TCO-PEG**_**4**_ (69,400 M^–1^ s^–1^), only <1% of intact **2Pyr**_**2**_ was detected at the end of the experiment, in comparison to >90% of **DHP**_**2**_ (Figure 7b). In applications that require an extended stability of Tz (>10 h), **DHP**_**2**_ thus outperforms even highly reactive **2Pyr**_**2**_ (Figure S5). Overall, these results confirm that non-electron-withdrawing DHP substituents can be used to significantly increase the IEDDA reactivity of Tz while maintaining a high compound stability (Figure 7c).

## Conclusions

Our detailed investigation of the reactions of pyridyl-Tz with TCOs uncovered the key role of reduced Tz distortion energies caused by repulsive intramolecular interactions. Based on these insights, we have been able to confirm an analogous effect in the case of vinyl ether-Tz and showed that 3,4-dihydro-2*H*-pyran (DHP) substitution increases IEDDA reactivity without accelerating Tz degradation under physiological conditions. Overall, we provide a new mechanistic understanding that may be instrumental in the rational design of next-generation bioorthogonal tools with enhanced reactivity and stability, particularly for strategies that require or benefit from long-term Tz stability.^9,50−52^

## Supporting Information Available

Computational methods, organic synthesis, reaction kinetics, compound stability and characterization, and copies of NMR spectra (PDF)
Coordinates of all calculated geometries (ZIP)
