# Supplementary material for: Uncovering the Key Role of Distortion in Bioorthogonal Tetrazine Tools That Defy the Reactivity/Stability Trade-Off
Source: J Am Chem Soc. 2022 May 2;144(18):8171–7. doi: 10.1021/jacs.2c01056 (PMC9100665; doi:10.1021/jacs.2c01056)
Supplement: Supplementary file 1 — ja2c01056_si_001.pdf [file ja2c01056_si_001.pdf]

## Supporting Information

# Uncovering the Key Role of Distortion in Bioorthogonal Tetrazine Tools that Defy the Reactivity/Stability Tradeoff

Dennis Svatunek, [†,⊥,*] Martin Wilkovitsch, [†,⊥] Lea Hartmann,[†] K. N. Houk,[‡] and Hannes Mikula[†,*]

[†]Institute of Applied Synthetic Chemistry, TU Wien, 1060 Vienna, Austria

[‡]Department of Chemistry and Biochemistry, University of California, Los Angeles, Los Angeles, 90095, USA

[⊥]D.S. and M.W. contributed equally

[*]Corresponding authors: dennis.svatunek@tuwien.ac.at, hannes.mikula@tuwien.ac.at

### Table of contents

1) Computational methods and results ............................................... S2
2) Synthesis ............................................................................... S5
3) Reactions kinetics................................................................... S8
4) Stability and solubility of tetrazines .......................................... S9
5) NMR spectra.......................................................................... S11
6) References ............................................................................ S16

# 1) Computational methods and results

Theoretical calculations were performed using the software package Gaussian16 RevC.01.[1] Geometry optimizations and frequency analyses were performed using the ωB97X-D density functional[2] and the 6-311G(d,p) basis set in 1,4-dioxane using the SMD[3] model. A quasi-harmonic correction to entropy was applied by setting all frequencies below 100 $cm^{-1}$ to 100 $cm^{-1}$ using GoodVibes.[4] All possible conformers were considered. Single imaginary frequencies corresponding to the desired reaction coordinates were obtained only in the case of transition state (TS) calculations. No imaginary frequencies were obtained for all other structures. Orbital energies were calculated at the HF/6-311+G(d,p)-SMD(1,4-dioxane)//wB97X-D/6-311G(d,p)-SMD(1,4-dioxane) level of theory. Distortion/interaction analyses were performed using autoDIAS.[5] The calculated energies are summarized in Table S1. Coordinates of all optimized geometries are provided as *.xyz files.

**Table S1**. wB97X-D/6-311G(d,p)-SMD(1,4-dioxane) calculated energies for all investigated Tz and the transition states for the reactions with **TCO** and ethylene.

| Structure | ΔE (hartree) | ZPE (hartree) | $\Delta H_{298}$ (hartree) | $\Delta G_{298}$ (hartree) |
|---|---|---|---|---|
| **TCO** | -313.232583 | 0.204800 | -313.019344 | -313.058930 |
| **Ph** | -527.320368 | 0.133779 | -527.177034 | -527.220506 |
| **2Pyr** | -543.349967 | 0.121652 | -543.218841 | -543.262222 |
| **3Pyr** | -543.352935 | 0.121878 | -543.221578 | -543.264956 |
| **4Pyr** | -543.352053 | 0.121656 | -543.220910 | -543.264345 |
| **MVE** | -412.997911 | 0.113361 | -412.875847 | -488.112664 |
| **MV** | -412.997911 | 0.113361 | -412.875847 | -412.916845 |
| **$TS_{Ph\_TCO}$** | -840.550682 | 0.340848 | -840.192584 | -840.251534 |
| **$TS_{2Pyr\_TCO}$** | -856.584023 | 0.328994 | -856.237907 | -856.296723 |
| **$TS_{3Pyr\_TCO}$** | -856.583923 | 0.329152 | -856.237621 | -856.296451 |
| **$TS_{4Pyr\_TCO}$** | -856.584047 | 0.329015 | -856.237860 | -856.296762 |
| **$TS_{MVE\_TCO}$** | -801.430038 | 0.325624 | -801.087097 | -801.146087 |
| **$TS_{MV\_TCO}$** | -726.227561 | 0.320584 | -725.890618 | -725.947504 |
| **ethylene** | -78.580105 | 0.051159 | -78.524966 | -78.551118 |
| **$TS_{Ph\_ethylene}$** | -605.881285 | 0.188576 | -605.681211 | -605.728542 |
| **$TS_{2Pyr\_ethylene}$** | -621.914597 | 0.176552 | -621.726632 | -621.773951 |
| **$TS_{3Pyr\_ethylene}$** | -621.914324 | 0.176797 | -621.726137 | -621.773359 |
| **$TS_{4Pyr\_ethylene}$** | -621.914469 | 0.176605 | -621.726439 | -621.773758 |

a

| Tz | LUMO+1 (eV) |
|---|---|
| **Ph** | -0.02 |
| **2Pyr** | -0.61 |
| **3Pyr** | -0.67 |
| **4Pyr** | -0.86 |

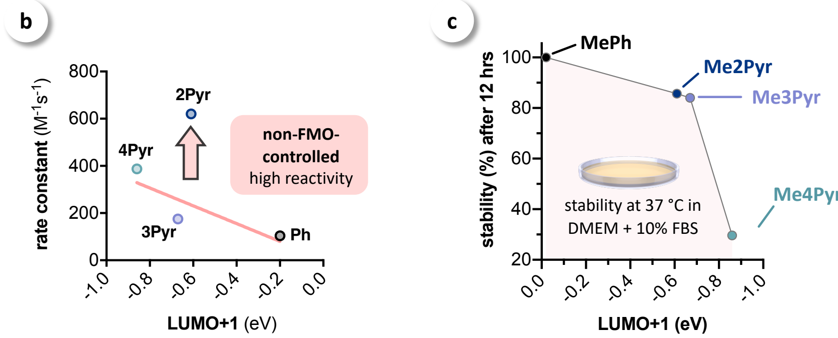


**Figure S1**. (a) Calculated Kohn-Sham LUMO+1 energies (ωB97X-D/6-311G(d,p)-SMD(1,4-dioxane)); (b) Measured rate constants vs. calculated Kohn-Sham LUMO+1 energy (cf. HF orbital energies in Fig. 2c); (c) Stability of Tz in cell growth medium at 37 °C (n = 3, SD < 5%) vs. Kohn-Sham LUMO+1 energy (cf. HF orbital energies in Fig. 5b).

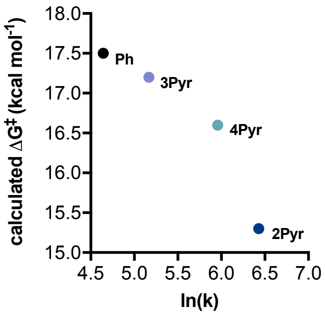


**Figure S2**. Calculated free energies of activation (ΔG‡) vs. the natural logarithm of the observed second order rate constants (k) for the IEDDA reactions of **Ph**, **2Pyr**, **3Pyr**, and **4Pyr** with **TCO**[6].

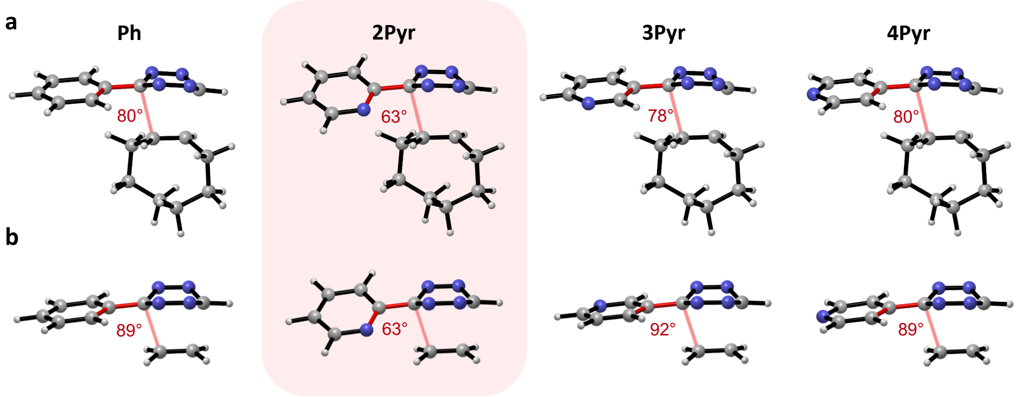


**Figure S3**. Dihedral angle in the TS for the reaction of **Ph**, **2Pyr**, **3Pyr**, and **4Pyr** with (a) **TCO** and (b) ethylene.

### Distortion/interaction analysis at consistent geometry

Different asynchronicity at the transition state can lead to skewed results in a distortion/interaction analysis. To verify the results as shown in Fig. 3b we performed the analysis using a consistent length of forming bonds for the IEDDA reaction of **Ph**, **2Pyr**, **3Pyr**, and **4Pyr** with **TCO**. At the transition state **2Pyr** shows a slightly more synchronous transition state with one bond being 0.03 to 0.04 Å longer and the other bond being 0.02 to 0.03 Å shorter than for the other tetrazines. Even at the transition state all bond lengths are within 0.04 Å. For the consistent geometry the bond lengths were set to 2.15 Å and 2.32 Å, respectively. Table S2 summarizes the results of the distortion/interaction analysis at the transition state and at the selected consistent geometry. While the calculated values vary slightly, the observed trends are very similar in both cases. In particular, the calculated distortion energy ($\Delta E_{dist}$) for **2Pyr** is considerably lower than the value obtained for **4Pyr**, indicating the key role of $\Delta E_{dist}$ regarding the high reactivity of 2-pyridyl-substituted tetrazines.

**Table S2**. Distortion/interaction analysis at the transition state and consistent geometry.

| Tz | Transition state | | | Consistent geometry | | |
|---|---|---|---|---|---|---|
| | ΔE (kcal/mol) | $\Delta E_{dist}$ (kcal/mol) | $\Delta E_{int}$ (kcal/mol) | ΔE (kcal/mol) | $\Delta E_{dist}$ (kcal/mol) | $\Delta E_{int}$ (kcal/mol) |
| **Ph** | 1.8 | 18.8 | -17.0 | 1.8 | 18.2 | -16.5 |
| **2Pyr** | -1.0 | **17.1** | -18.1 | -0.9 | **17.9** | -18.9 |
| **3Pyr** | 1.4 | 18.7 | -17.3 | 1.4 | 18.6 | -17.2 |
| **4Pyr** | 0.8 | **18.5** | -17.8 | 0.8 | **19.0** | -18.3 |

### NBO analysis

To further investigate the interaction between **2Pyr** and **TCO** at the transition state, an interaction analysis using NBO 6.0[7] was performed, identifying two additional interactions that are enabled by the rotation of the pyridyl group: (1) a weak hydrogen bond between the pyridyl nitrogen lone pair and the vinylic CH bond of **TCO** with a second-order perturbation energy of 0.37 kcal mol$^{-1}$ (Fig. S4a); (2) an $n_N \rightarrow \sigma^*_{C\text{-}N}$ interaction between the pyridyl lone pair and one of the C-N bonds in the tetrazine, which is already present in the reactant (0.33 kcal mol$^{-1}$) and increases to 0.60 kcal mol$^{-1}$ in the transition state (Fig. S4b).

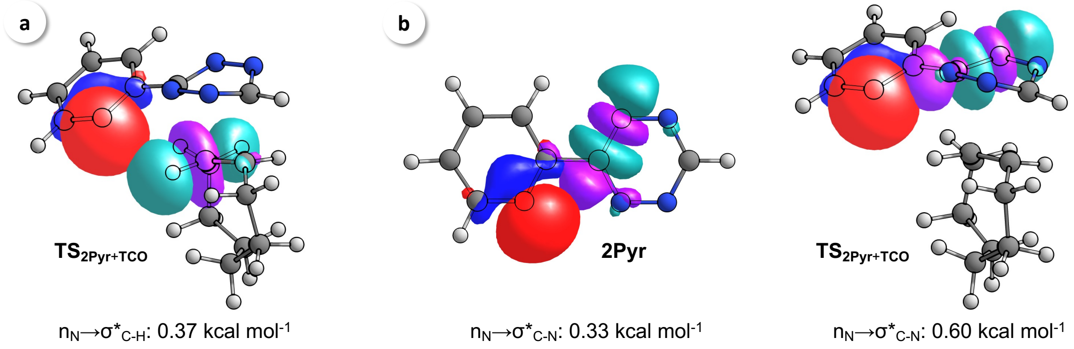


**Figure S4**. (a) NBO analysis of the intermolecular hydrogen bond at the transition state (**2Pyr+TCO**). (b) NBO analysis of the intramolecular $n_N \rightarrow \sigma^*_{C\text{-}N}$ interaction in the reactant (**2Pyr**, left) and the transition state (right). Bonding orbital shown in red/blue, antibonding in purple/teal.

## 2) Synthesis

### General methods

Unless otherwise noted, reactions were carried out under an atmosphere of argon in air-dried glassware with magnetic stirring. Air- and/or moisture-sensitive liquids were transferred via syringe. All reagents were purchased from commercial sources without further purification. Dichloromethane (DCM) was dried using PURESOLV-columns (Inert Corporation, USA). Solvents used for flash column chromatography were purchased from Donau Chemie AG (Austria). Dry acetonitrile was commercially obtained from Sigma-Aldrich (Germany) and stored under argon. Column chromatography was performed using a BUCHI Sepacore Flash System (2 x BUCHI Pump Module C-605, BUCHI Pump Manager C-615, BUCHI UV Photometer C-635, and BUCHI Fraction Collector C-660) and a Reveleris® X2 Flash Chromatography/Prep Purification Systems (BUCHI). Silica gel 60 (40-63 µm) was obtained from Merck. A Kinetex® 5 µm C18 100 Å, AXIA LC column (100 x 30.0 mm, Phenomenex) was used for preparative HPLC. HPLC grade solvents were purchased from VWR (USA). $^1$H and $^{13}$C NMR spectra were recorded on a Bruker AC 200 MHz, Bruker Avance UltraShield 400 MHz or Bruker Ascend 600 MHz spectrometer at 20 °C. Chemical shifts are reported in ppm (δ) relative to tetramethylsilane and calibrated using solvent residual peaks. Data is shown as follows: Chemical shift, multiplicity (s = singlet, d = doublet, t = triplet, q = quartet, quint = quintet, m = multiplet, b = broad signal), coupling constants (*J*, Hz) and integration. HRMS analysis was carried out using methanol solutions (concentration: 10 ppm) on an Agilent 6230 LC TOFMS mass spectrometer equipped with an Agilent Dual AJS ESI-Source. The mass spectrometer was connected to a liquid chromatography system of the 1100/1200 series from Agilent Technologies (Palo Alto, CA, USA).

### General procedure A for the synthesis of H-aryl-Tz

To a mixture of nitrile (1 eq.), sulfur (2 eq.), ethanol (15 eq.) and dichloromethane (1 eq.) in a microwave-vial was added hydrazine monohydrate (8 eq.) and the vial was sealed subsequently. The reaction mixture was stirred at 60 °C for the time specified after which it was cooled in an ice-bath and a solution of sodium nitrite (6.5 eq.) in water (10 mL) was added followed by addition of acetic acid (9 mL). The resulting colorful solution was extracted with DCM (4 x 100 mL), the combined organic layer was dried over $Na_2SO_4$, filtered, and concentrated. The crude product was purified by column chromatography.

### 3-Phenyl-1,2,4,5-tetrazine (Ph)

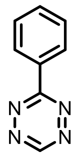


Synthesis was carried out according to general procedure A using benzonitrile (200 mg, 1.94 mmol), sulfur (124.4 mg, 3.88 mmol), ethanol (1.7 mL, 29 mmol), dichloromethane (124 µL, 1.94 mmol) and hydrazine monohydrate (777 µL, 15.52 mmol) for 21 h. After oxidation, purification was performed by preparative column chromatography (Silica 100A, Phenomenex Luna 10u, 250 x 21.2 mm, 10 µm, 0-10% EtOAc in hexanes, gradient elution) to give the desired product as a pink solid (17 mg, 6%). Spectroscopic data matched that reported previously.[8]

### 3-(2-Pyridyl)-1,2,4,5-tetrazine (2Pyr)

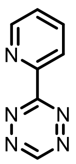


Synthesis was carried out according to general procedure A using 2-pyridinecarbonitrile (200 mg, 1.92 mmol), sulfur (123.1 mg, 3.84 mmol), ethanol (1.7 mL, 29 mmol), dichloromethane (122 µL, 1.92 mmol) and hydrazine monohydrate (744 µL, 15.36 mmol) for 21 h. After oxidation, purification was performed by column chromatography (50-90% EtOAc in hexanes, gradient elution) to give the desired product as a pink solid (47 mg, 15%). Spectroscopic data matched that previously reported.[9]

### 3-(3-Pyridyl)-1,2,4,5-tetrazine (3Pyr)

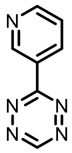

Synthesis was carried out according to general procedure A using 3-pyridinecarbonitrile (200 mg, 1.92 mmol), sulfur (123.1 mg, 3.84 mmol), ethanol (1.7 mL, 29 mmol), dichloromethane (122 µL, 1.92 mmol) and hydrazine monohydrate (744 µL, 15.36 mmol) for 21 h. After oxidation, purification was performed by column chromatography (40-70% EtOAc in hexanes, gradient elution) to give the desired product as a pink solid (78 mg, 26%); $^{1}H$ NMR (600 MHz, $CDCl_3$) δ 10.32 (s, 1H), 9.83–9.86 (m, 1H), 8.99–8.92 (m, 2H), 7.60–7.56 (m, 1H); $^{13}C$ NMR (151 MHz, $CDCl_3$) δ 165.64, 158.41, 153.75, 149.64, 135.69, 127.86, 124.22; HRMS $[M+H]^+$ m/z calcd. 160.0618 for $C_7H_6N_5^+$, found 160.0615.

### 3-(4-Pyridyl)-1,2,4,5-tetrazine (4Pyr)

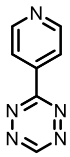

Synthesis was carried out according to general procedure A using 4-pyridinecarbonitrile (200 mg, 1.92 mmol), sulfur (123.1 mg, 3.84 mmol), ethanol (1.7 mL, 29 mmol), dichloromethane (122 µL, 1.92 mmol) and hydrazine monohydrate (744 µL, 15.36 mmol) for 20 h. After oxidation, purification was performed by preparative column chromatography (Silica 100A, Phenomenex Luna 10u, 250 x 21.2 mm, 10 µm, 20-40% EtOAc in hexanes, gradient elution) to afford the desired product as a pink solid (17 mg, 6%); $^{1}H$ NMR (600 MHz, $CDCl_3$) δ 10.36 (s, 1H), 8.95 (dd, *J* = 6.1, 2.7 Hz, 2H), 8.5 (dd, *J* = 6.1, 2.8 Hz, 2H); $^{13}C$ NMR (151 MHz, $CDCl_3$) δ 165.53, 158.76, 151.16, 139.33, 121.71; HRMS $[M+H]^+$ m/z calcd. 160.0618 for $C_7H_6N_5^+$, found 160.0615.

## General procedure B for the synthesis of methyl-aryl-Tz

To a well-blended mixture of nitrile (1 eq.), acetonitrile (5-7 eq.) and catalytic amounts of $NiCl_2$ or $Zn(OTf)_2$ (0.25-0.3 eq.) was added hydrazine monohydrate (10-25 eq.). The reaction mixture was stirred at 60 °C for 20 h after which it was cooled in an ice-bath and a solution of sodium nitrite (6.5 eq.) in water (10 mL) was added followed by the addition of acetic acid or aqueous 2N HCl. The resulting colorful solution was extracted with DCM (4 x 100 mL), the combined organic layer was dried over $Na_2SO_4$, filtered, and concentrated. The crude product was purified by column chromatography.

### 3-Methyl-6-phenyl-1,2,4,5-tetrazine (MePh)

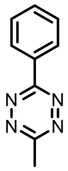

Synthesis was carried out according to general procedure B using benzonitrile (6 g, 58 mmol), acetonitrile (16.7 g, 408 mmol), $NiCl_2$ (0.3 g, 3 mmol) and hydrazine monohydrate (72 g, 1.5 mol). After oxidation, purification was performed by column chromatography (EtOAc in hexanes, gradient elution) to give the desired product as a pink solid (420 mg, 4%). Spectroscopic data matched that previously reported.[8]

### 3-Methyl-6-(2-pyridyl)-1,2,4,5-tetrazine (Me2Pyr)

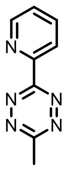

Synthesis was carried out according to general procedure B using 2-pyridinecarbonitrile (250 mg, 2.4 mmol), acetonitrile (627 µL, 12 mmol), $Zn(OTf)_2$ (218 mg, 0.6 mmol) and hydrazine monohydrate (1.4 mL, 28.8 mmol). After oxidation, purification was performed by column chromatography (40-80% EtOAc in hexanes, gradient elution) to give the desired product as a pink solid (83 mg, 20%). Spectroscopic data matched that previously reported.[10]

### 3-Methyl-6-(3-pyridyl)-1,2,4,5-tetrazine (Me3Pyr)

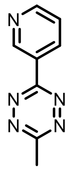

Synthesis was carried out according to general procedure B using 3-pyridinecarbonitrile (500 mg, 4.8 mmol), acetonitrile (1.3 mL, 24 mmol), $Zn(OTf)_2$ (436 mg, 1.2 mmol) and hydrazine monohydrate (2.8 mL, 57.6 mmol). After oxidation, purification was performed by column chromatography (50-90% EtOAc in hexanes, gradient elution) to afford the desired product as a purple solid (37 mg, 4%). Spectroscopic data matched that previously reported.[11]

### 3-Methyl-6-(4-pyridyl)-1,2,4,5-tetrazine (Me4Pyr)

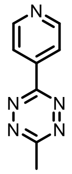

Synthesis was carried out according to general procedure B using 4-cyanopyridine (540 mg, 5.2 mmol), acetonitrile (1.1 mL, 26.8 mmol), $Zn(OTf)_2$ (473 mg, 1.3 mmol) and hydrazine monohydrate (2.5 g, 50 mmol). After oxidation, purification was performed by column chromatography (5%-40% EtOAc in hexanes, gradient elution) to afford the desired product as a purple solid (125 mg, 14%). Spectroscopic data matched that previously reported.[11]

## Synthesis of vinylether-substituted Tz

### 3-Methyl-6-(1-ethoxyvinyl)-1,2,4,5-tetrazine (MeEVE)

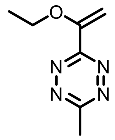

Synthesis was performed similar to a known procedure.[12] A mixture of 3-([1,1'-Biphenyl]-4-ylmethylthio)-6-methyl-1,2,4,5-tetrazine[13] (75 mg, 0.25 mmol), tributyl(1-ethoxyvinyl)tin (172 µL, 0.51 mmol), CuTc (97 mg, 0.51 mmol) and $Pd(PPh)_3$ (44 mg, 40 µmol) in dry dioxane (51 mL) was stirred at 100 °C for 20 min. Purification by column chromatography (10% $K_2CO_3$ modified silica, 7-75% DCM in hexanes, gradient elution) gave the desired product as a pink solid (10.2 mg, 24%); $^1$H NMR (400 MHz, $CD_2Cl_2$) δ 5.87 (d, *J* = 2.8 Hz, 1H), 4.86 (d, *J* = 2.8 Hz, 1H), 4.08 (q, *J* = 7.0 Hz, 2H), 3.03 (s, 3H), 1.48 (t, *J* = 7.0 Hz, 3H); $^{13}$C NMR (101 MHz, $CD_2Cl_2$) δ 168.1, 162.3, 154.3, 92.7, 64.9, 21.4, 14.5.

Note: **MeEVE** was stored as 5 mM solution in $CD_2Cl_2$ in -20°C to prevent self-condensation.[12]

### 3-Methyl-6-(3,4-dihydro-2*H*-pyran-6-yl)-1,2,4,5-tetrazine (MeDHP)

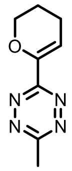

Synthesis was performed analogously to **MeEVE** using 3-([1,1'-biphenyl]-4-ylmethylthio)-6-methyl-1,2,4,5-tetrazine[13] (75 mg, 0.25 mmol), tributyl(5,6-dihydro-4*H*-pyran-2-yl)stannane (170 µL, 0.51 mmol), CuTc (97 mg, 0.51 mmol) and $Pd(PPh)_3$ (44 mg, 40 µmol) in dry dioxane (51 mL). The reaction mixture was stirred at 100 °C for 26 min. Purification by preparative column chromatography (Silica 100 A, Phenomenex Luna 10u, 250 x 21.2 mm, 10 µm, 0-1% DCM in MeOH) gave the desired product as a red oil (4.9 mg, 11%); $^1$H NMR (600 MHz, $CDCl_3$) δ 6.69 (t, *J* = 4.2 Hz, 1H), 4.35 (t, *J* = 5.1 Hz, 2H), 3.05 (s, 3H), 2.39 (q, *J* = 4.6 Hz, 2H), 2.04 (quint, *J* = 5.3 Hz, 2H); $^{13}$C NMR (151 MHz, $CDCl_3$) δ 167.3, 161.2, 146.7, 110.3, 67.3, 21.9, 21.3, 21.2.

### 3,6-Bis(3,4-dihydro-2*H*-pyran-6-yl)-1,2,4,5-tetrazine ($DHP_2$)

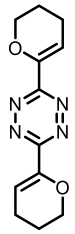

Synthesis was performed analogously to **MeEVE** using 3,6-bis(methylthio-1,2,4,5-tetrazine (44 mg, 0.25 mmol), tributyl(5,6-dihydro-4*H*-pyran-2-yl)stannane (340 µL, 1.0 mmol), CuTc (192 mg, 1.0 mmol) and $Pd(PPh)_3$ (44 mg, 40 µmol) in dry dioxane (51 mL). The reaction mixture was stirred at 100 °C for 65 min. Purification by preparative column chromatography (Silica 100A, Phenomenex Luna 10u, 250 x 21.2 mm, 10 µm, 0-20% DCM in MeOH, gradient elution) gave the desired product as red crystals (12.8 mg, 21%); $^1$H NMR (600 MHz, $CDCl_3$) δ 6.71 (t, *J* = 4.3 Hz, 2H), 4.34 (t, *J* = 5.0 Hz, 4H), 2.39 (q, *J* = 4.7 Hz, 4H), 2.03 (quint, *J* = 5.1 Hz, 4H); $^{13}$C NMR (151 MHz, $CDCl_3$) δ 160.9, 146.8, 110.7, 67.2, 21.9, 21.3.

## 3) Reaction kinetics

### Sample preparation

**TCO**[6] was dissolved in dry 1,4-dioxane to reach a concentration of 2 mM.
An approx. 20 mM stock solution of **TCO-PEG$_4$** [14] in DMSO was prepared. The exact concentration was determined by absorbance titration with DMT (extinction coefficient 510 $M^{-1}cm^{-1}$ at 520 nm), quantifying the decrease in tetrazine absorbance upon reaction with TCO. The initial DMSO stock was diluted with Dulbecco's Phosphate Buffered Saline (DPBS) to prepare solutions for stopped-flow analysis at a final TCO concentration of 1 mM.
Tetrazines **Ph**, **2Pyr**, **3Pyr**, and **4Pyr** were dissolved in dry 1,4-dioxane to reach a concentration of approx. 10 µM and used for stopped-flow analysis.
20 mM stock solutions of compounds **MePh**, **Me2Pyr**, **Me3Pyr**, **Me4Pyr**, **2Pyr$_2$**, **MeEVE**, **MeDHP**, and **DHP$_2$** in DMSO were prepared. Serial dilution into DPBS gave solutions for stopped-flow analysis at a tetrazine concentration of 100 µM.

### Stopped-flow spectrophotometry

Stopped-flow measurements were performed using an SX20-LED stopped-flow spectrophotometer (Applied Photophysics) equipped with a 535nm LED (optical pathlength 10 mm, full width half-maximum 34 nm) to monitor the characteristic tetrazine visible light absorbance (520-540 nm). The reagent syringes were loaded with tetrazine and TCO solutions and the instrument was primed. Measurements were done in sextuplicate for each tetrazine. Reactions were conducted at 25 °C or 37 °C and recorded automatically at the time of acquisition.

### Data analysis

Data sets were analyzed by fitting an exponential decay using Prism 6 (Graphpad) to calculate the observed pseudo-first order rate constants that were converted into second order rate constants (Table S3) by dividing through the concentration of excess TCO used.

**Table S3**. Determined second order rate constants (n = 6, SD < 1%).

| **Tetrazine** | Dienophile | Conditions | Second order rate constant ($M^{-1}s^{-1}$) |
|---|---|---|---|
| **Ph** | **TCO** | 1,4-dioxane, 25 °C | 100 |
| **2Pyr** | **TCO** | 1,4-dioxane, 25 °C | 620 |
| **3Pyr** | **TCO** | 1,4-dioxane, 25 °C | 175 |
| **4Pyr** | **TCO** | 1,4-dioxane, 25 °C | 387 |
| **MePh** | **TCO-PEG$_4$** | DPBS, 37 °C | 990 |
| **Me2Pyr** | **TCO-PEG$_4$** | DPBS, 37 °C | 5120 |
| **Me3Pyr** | **TCO-PEG$_4$** | DPBS, 37 °C | 1180 |
| **Me4Pyr** | **TCO-PEG$_4$** | DPBS, 37 °C | 2740 |
| **MeEVE** | **TCO-PEG$_4$** | DPBS, 37 °C | 2750 |
| **MeDHP** | **TCO-PEG$_4$** | DPBS, 37 °C | 1820 |
| **DHP$_2$** | **TCO-PEG$_4$** | DPBS, 37 °C | 6450 |
| **2Pyr$_2$** | **TCO-PEG$_4$** | DPBS, 37 °C | 69400 |

## 4) Stability and solubility of tetrazines

### Analytical stock solutions and solvents

The stability of tetrazines was assessed in full cell growth medium (DMEM (fluorobrite) + 10% FBS). Therefore, 20 mM stock solutions of all tetrazines (**Me2Pyr, Me3Pyr, Me4Pyr, MeEVE, MeDHP, DHP$_2$, 2Pyr$_2$**) in DMSO were prepared and further diluted into medium to reach a final concentration of 500 µM (DMSO content (v/v): 2.5%).

### Stability measurements

All tetrazine solutions were incubated in absorption cuvettes (1 mL) in triplicates at 37 °C. After 3, 6, 9, 12, and 24 h the tetrazine absorbance at 520 nm was measured on a Shimadzu UV1800 spectrophotometer with a temperature-controlled sample slide at 37 °C. After spectrometric analysis, incubation at 37 °C was continued and the procedure was repeated until the last measurement (24 h). The measured absorbance was compared to the absorbance at the initial value at 0 h to calculate the remaining fraction (%) of intact Tz (Table S4). At each time-point, freshly prepared Tz samples were measured to correct for variability of the measurement setup.

**Table S4**. Intact fraction of Tz (%) after incubation in DMEM + 10% FBS at 37 °C (n = 3, SD < 5%).

| Time (h) | MePh | Me2Pyr | Me3Pyr | Me4Pyr | MeEVE | MeDHP | DHP$_2$ | 2Pyr$_2$ |
|---|---|---|---|---|---|---|---|---|
| 3 | 97 | 95 | 100 | 86 | 99 | 97 | 97 | 69 |
| 6 | 98 | 93 | 97 | 68 | 98 | 96 | 95 | 40 |
| 9 | 99 | 87 | 93 | 49 | 97 | 94 | 93 | 16 |
| 12 | 100 | 80 | 88 | 34 | 94 | 94 | 92 | 5 |
| 24 | 99 | 46 | 70 | 13 | 82 | 92 | 91 | <1 |

In applications that require extended stability of the Tz (>10 h), **DHP$_2$** outperforms highly reactive **2Pyr$_2$**, even when using an excess of Tz (to compensate for degradation), as shown by the calculated apparent rate constants for the click reaction of pre-administered Tz (Fig. S5). In a similar analysis (taking into account stability and reactivity) the methyltetrazines **MePh**, **Me2Pyr**, **Me4Pyr**, **MeEVE**, and **MeDHP** have been compared (Fig. S6).

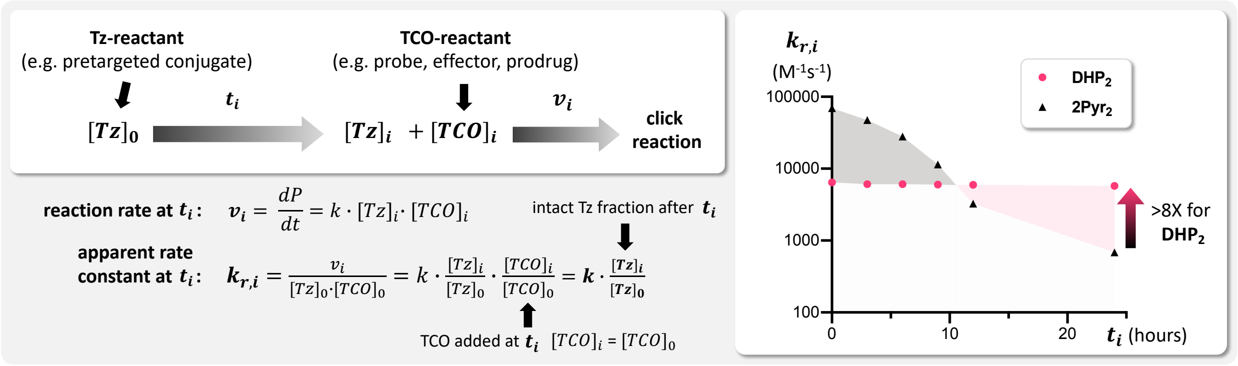

**Figure S5**. Calculation of apparent rate constants ($k_{r,i}$), taking into account the reactivity and stability of Tz.

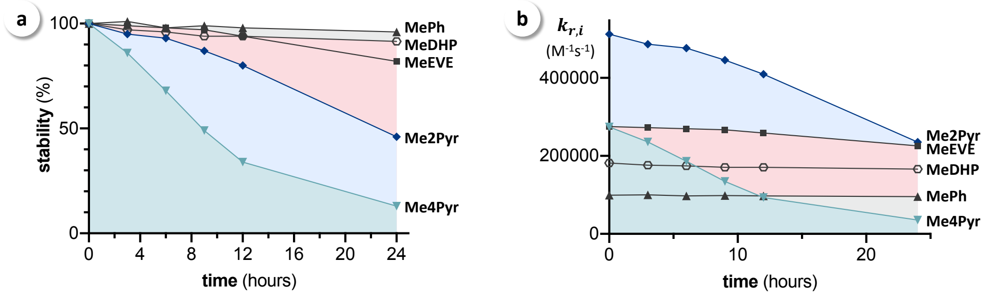


**Figure S6**. (a) Stability of the methyltetrazines **MePh**, **Me2Pyr**, **Me4Pyr**, **MeEVE**, and **MeDHP** under physiological conditions (full cell growth medium, 37 °C, n = 3, SD < 5%), and (b) apparent rate constants ($k_{r,i}$).

## Water solubility of tetrazines

To demonstrate the improved water solubility of vinyl ether-tetrazines, as indicated by data reported by Fox *et al.*,[12] we have prepared solutions of **MeEVE**, **MeDHP**, and **DHP$_2$** in water at a concentration of 2 mM. In contrast, **MePh** and **2Pyr$_2$** were not completely soluble at this level (Fig. S7), as suggested by calculated LogP values (cLogP, Chemicalize, ChemAxon).

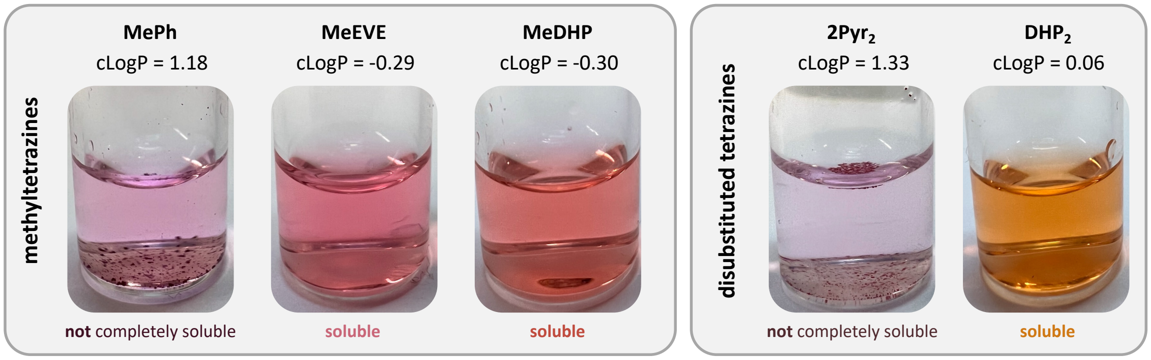


**Figure S7**. In contrast to **MePh** and **2Pyr$_2$**, the vinyl ether-tetrazines **MeEVE**, **MeDHP**, and **DHP$_2$** are completely soluble in water at a concentration of 2 mM.

## 5) NMR Spectra

Compound **3Pyr**, $^1$H NMR

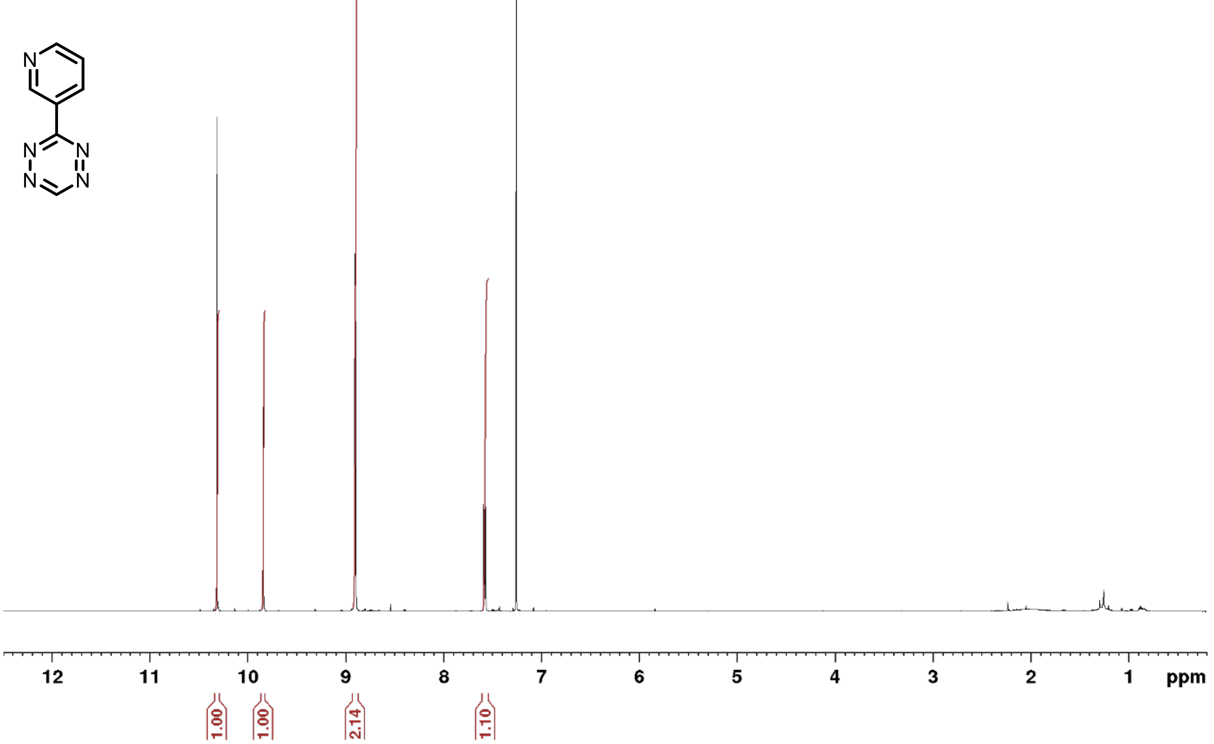


Compound **3Pyr**, $^{13}$C NMR

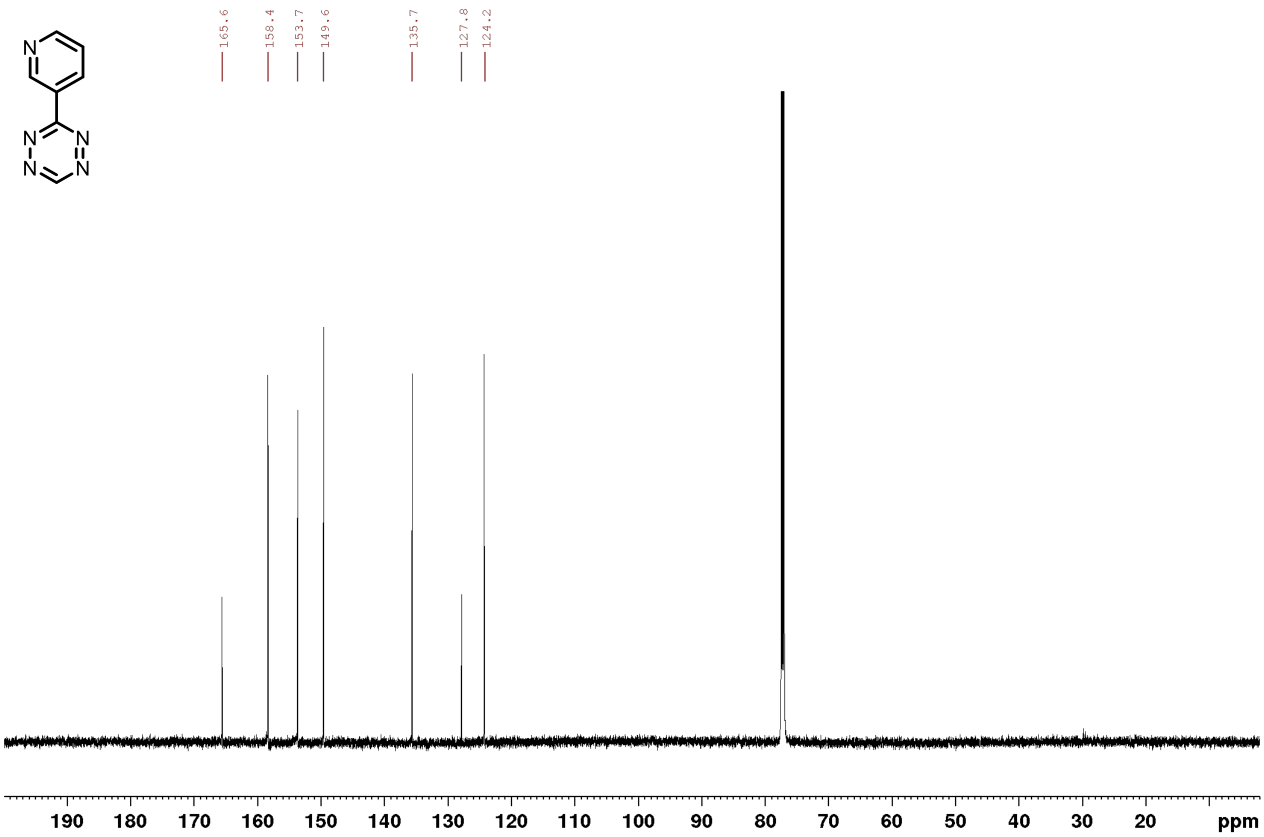

Compound **4Pyr**, $^{1}$H NMR

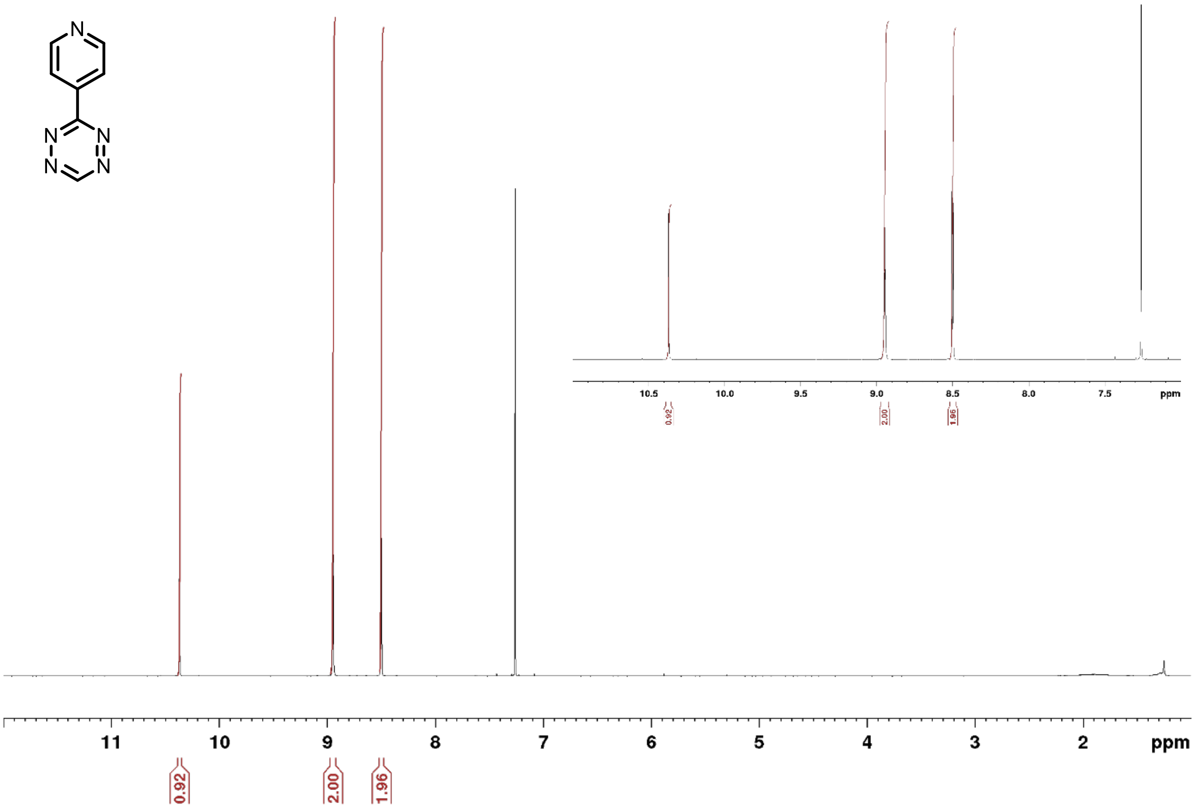


Compound **4Pyr**, $^{13}$C NMR

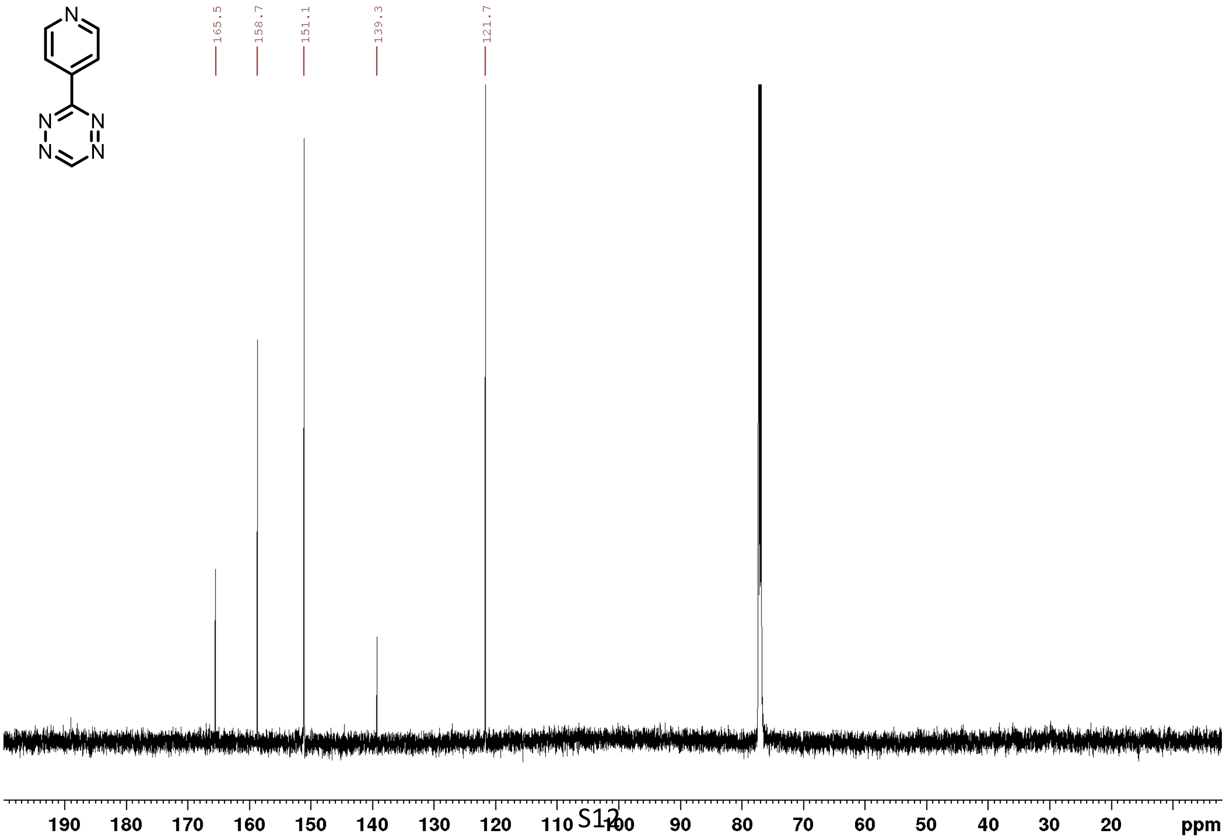

Compound **MeEVE**, $^{1}$H NMR

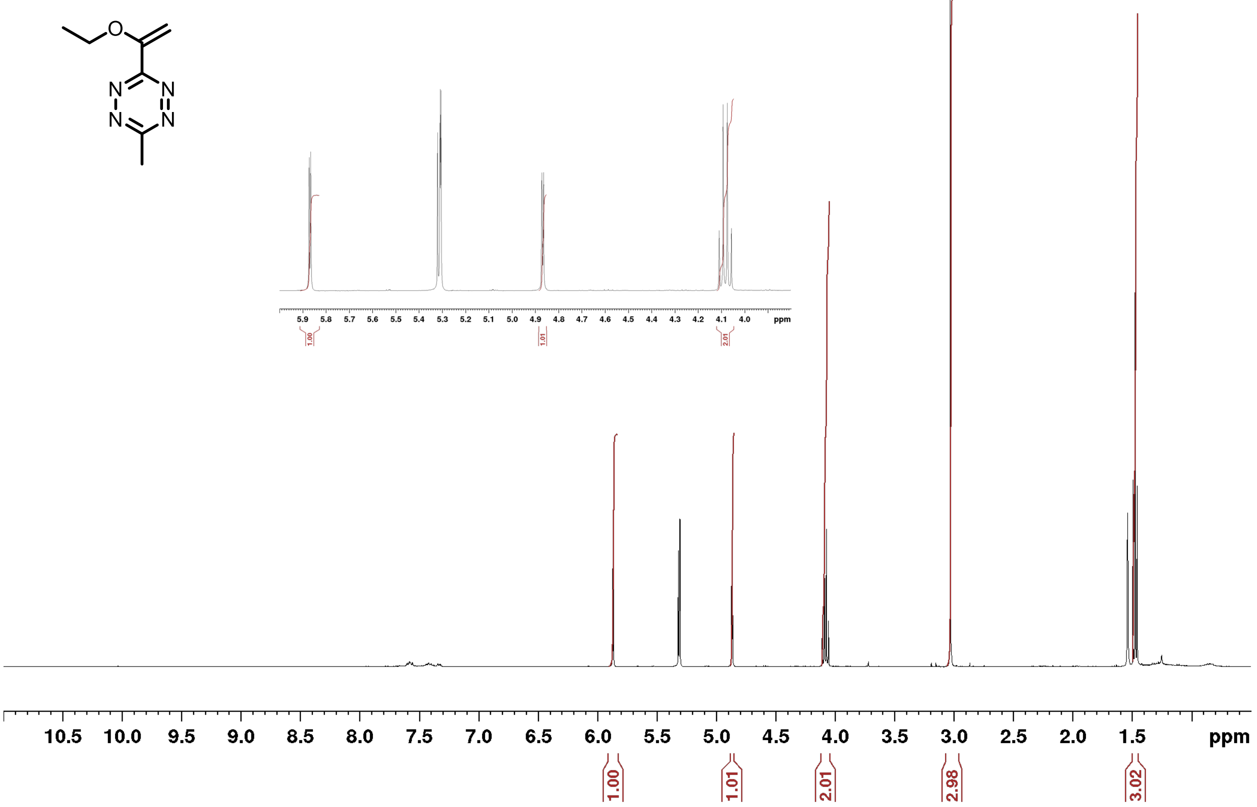


Compound **MeEVE**, $^{13}$C NMR

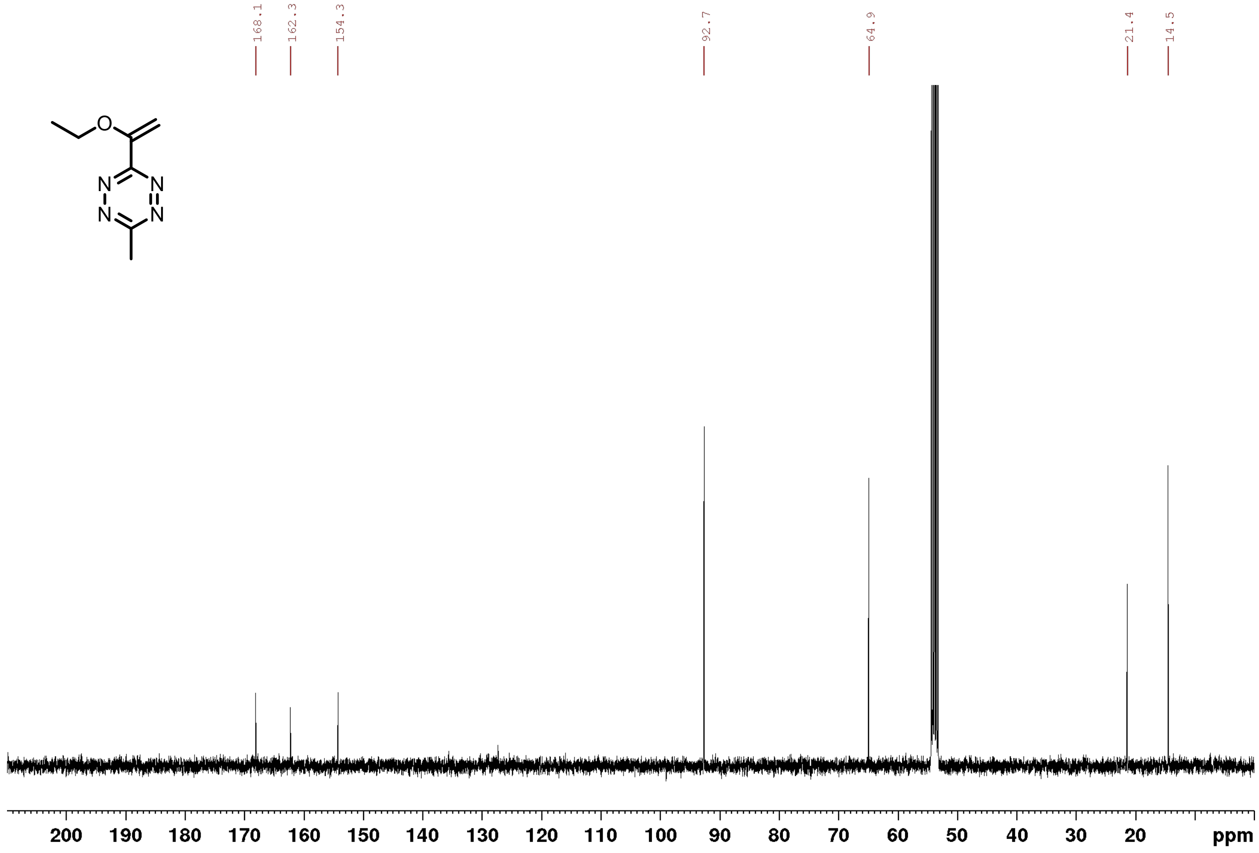

Compound **MeDHP**, $^{1}H$ NMR

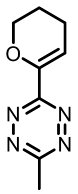

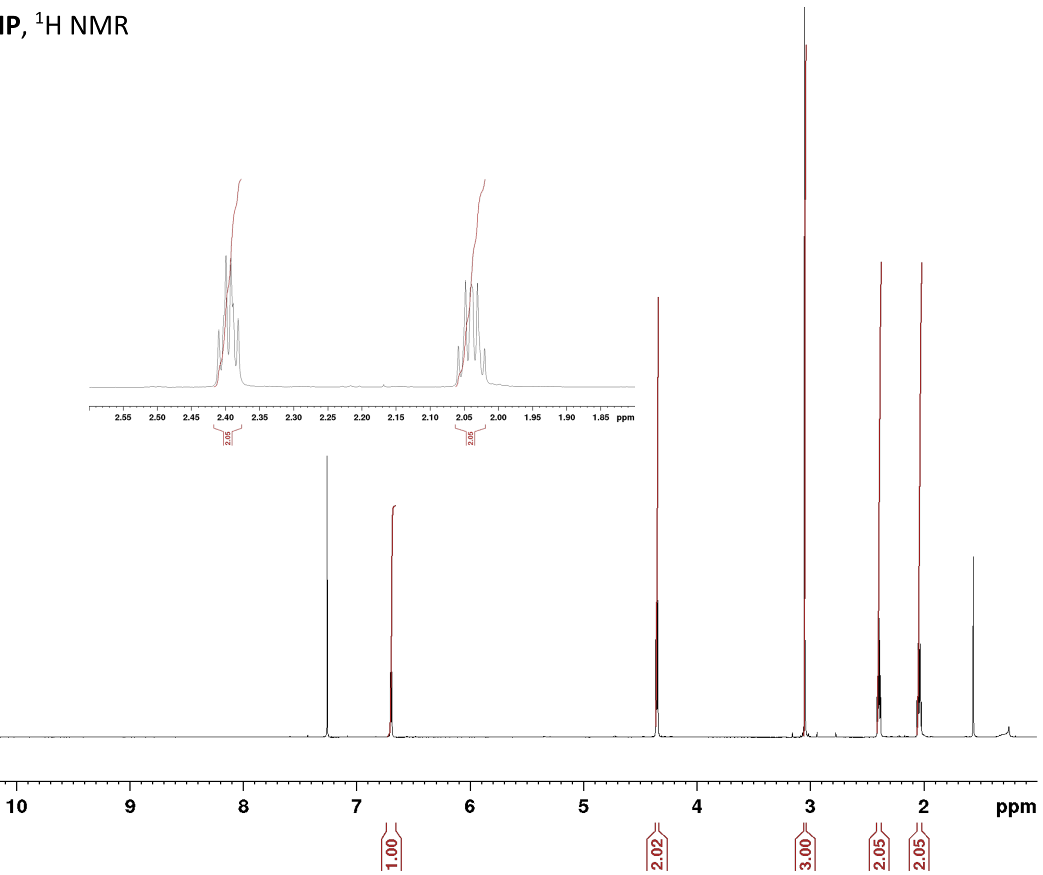


Compound **MeDHP**, $^{13}C$ NMR

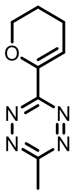

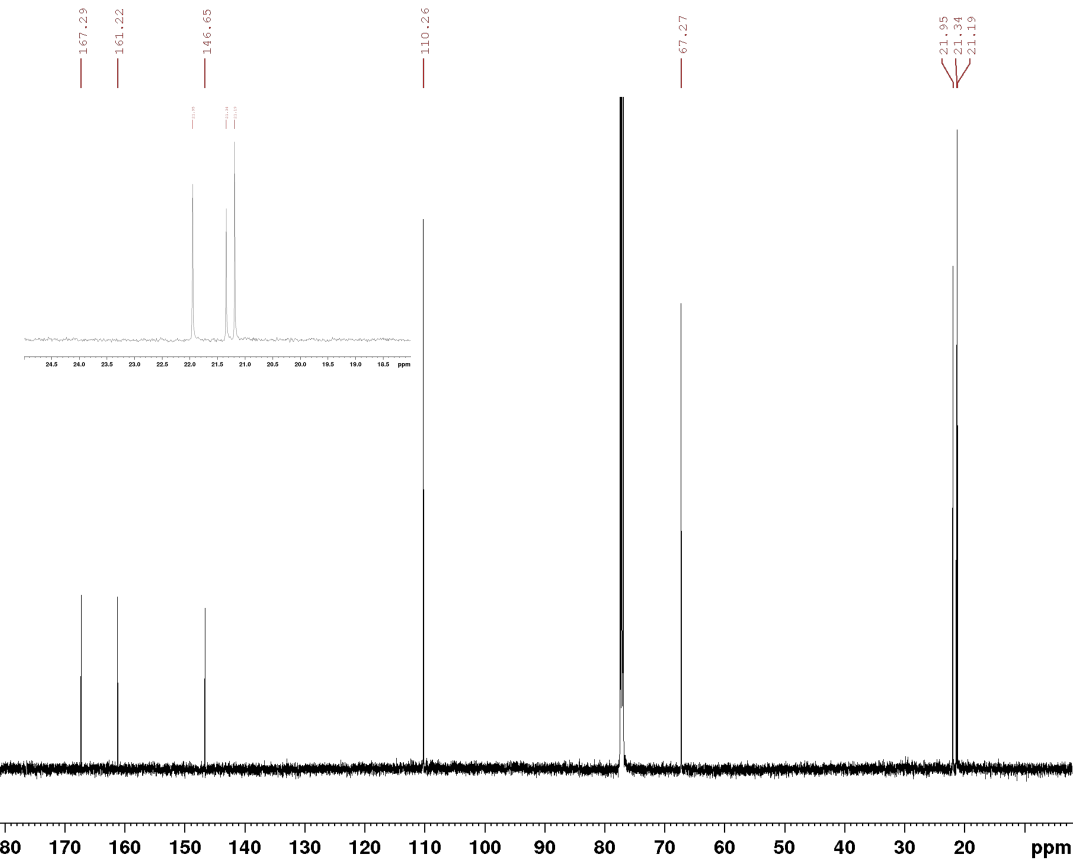

Compound **DHP$_2$**, $^1$H NMR

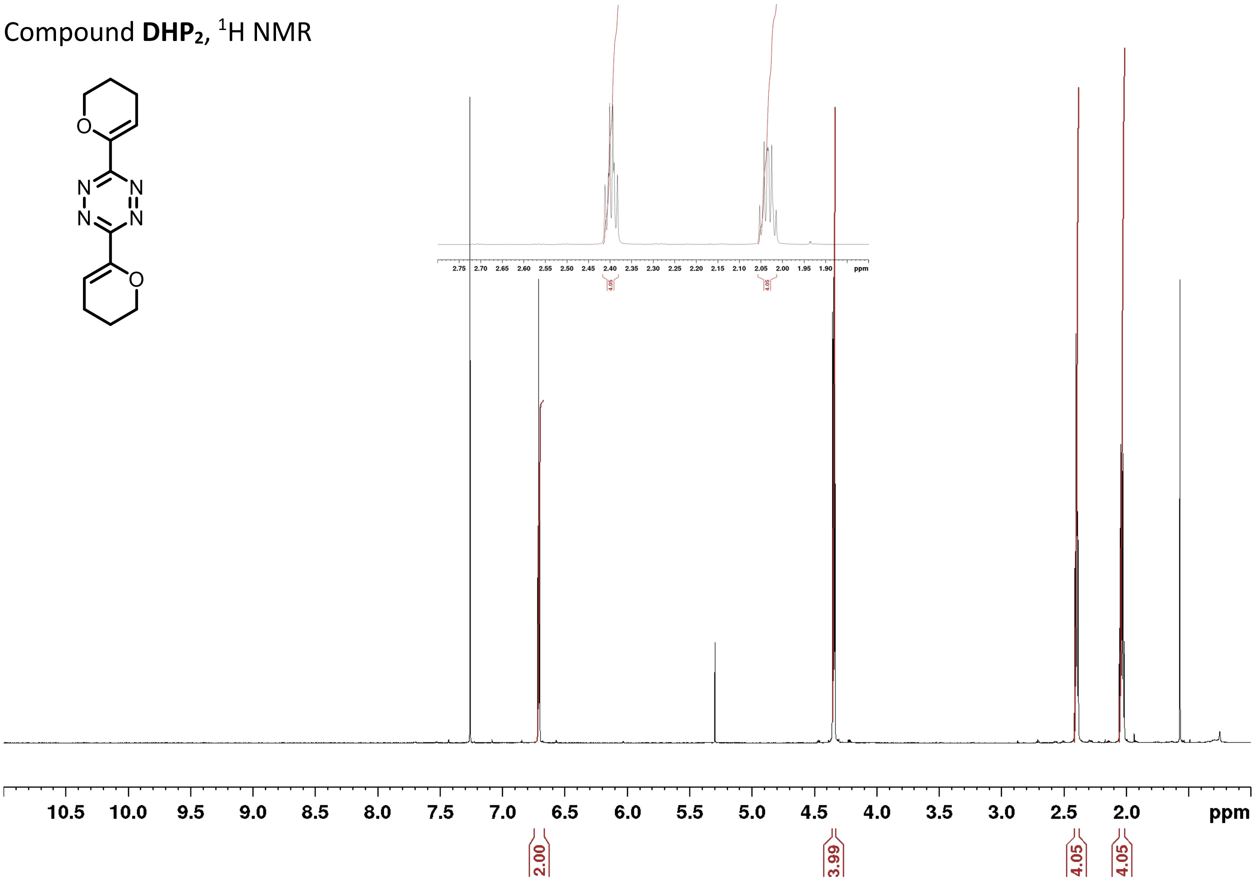


Compound **DHP$_2$**, $^{13}$C NMR

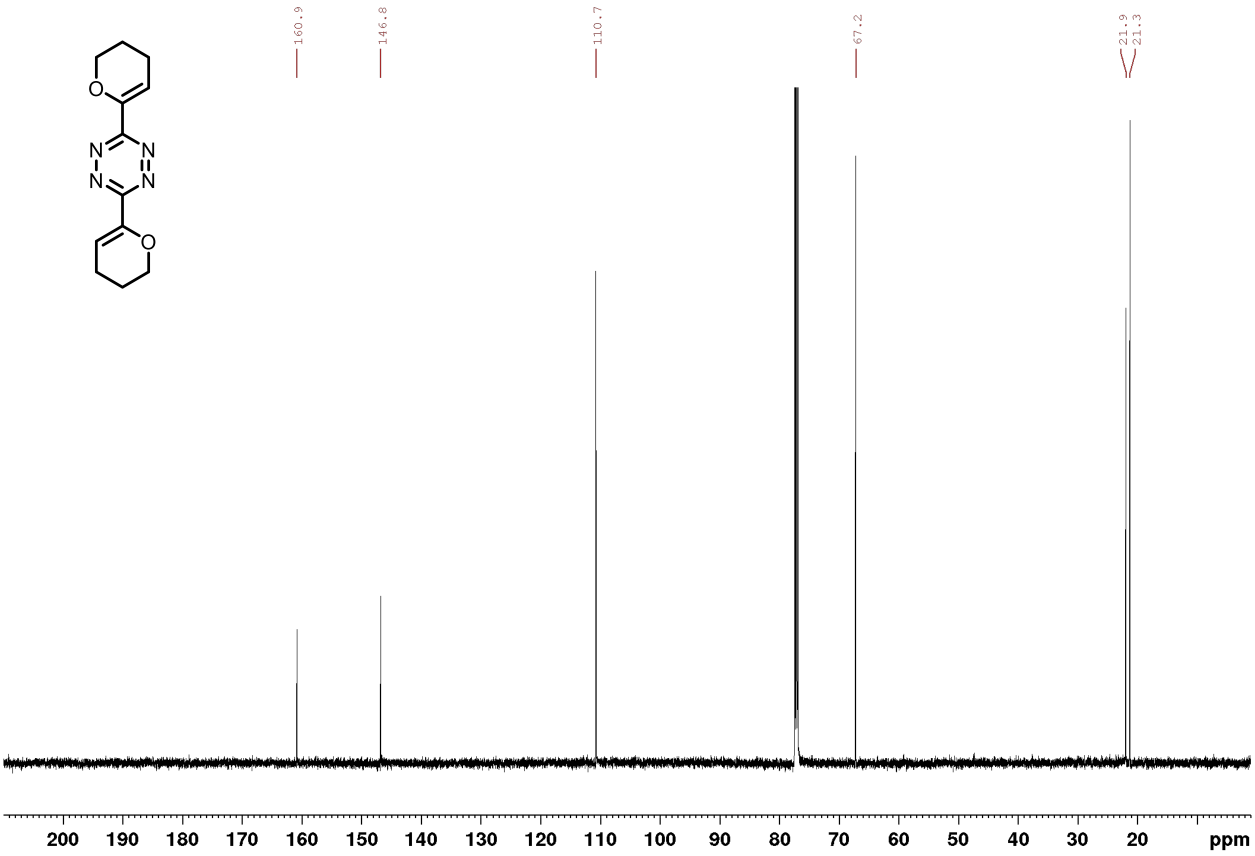

## 6) References

[1] Frisch, M. J.; Trucks, G. W.; Schlegel, H. B.; Scuseria, G. E.; Robb, M. A.; Cheeseman, J. R.; Scalmani, G.; Barone, V.; Petersson, G. A.; Nakatsuji, H.; Li, X.; Caricato, M.; Marenich, A. V.; Bloino, J.; Janesko, B. G.; Gomperts, R.; Mennucci, B.; Hratchian, H. P.; Ortiz, J. V.; Izmaylov, A. F.; Sonnenberg, J. L.; Williams; Ding, F.; Lipparini, F.; Egidi, F.; Goings, J.; Peng, B.; Petrone, A.; Henderson, T.; Ranasinghe, D.; Zakrzewski, V. G.; Gao, J.; Rega, N.; Zheng, G.; Liang, W.; Hada, M.; Ehara, M.; Toyota, K.; Fukuda, R.; Hasegawa, J.; Ishida, M.; Nakajima, T.; Honda, Y.; Kitao, O.; Nakai, H.; Vreven, T.; Throssell, K.; Montgomery Jr., J. A.; Peralta, J. E.; Ogliaro, F.; Bearpark, M. J.; Heyd, J. J.; Brothers, E. N.; Kudin, K. N.; Staroverov, V. N.; Keith, T. A.; Kobayashi, R.; Normand, J.; Raghavachari, K.; Rendell, A. P.; Burant, J. C.; Iyengar, S. S.; Tomasi, J.; Cossi, M.; Millam, J. M.; Klene, M.; Adamo, C.; Cammi, R.; Ochterski, J. W.; Martin, R. L.; Morokuma, K.; Farkas, O.; Foresman, J. B.; Fox, D. J. *Gaussian 16 Rev. C.01*, Wallingford, CT, 2016.

[2] Chai, J.-D.; Head-Gordon, M. Long-range corrected hybrid density functionals with damped atom–atom dispersion corrections. *Phys. Chem. Chem. Phys.* **2008**, *10*, 6615-6620.

[3] Marenich, A. V.; Cramer, C. J.; Truhlar, D. G. Universal Solvation Model Based on Solute Electron Density and on a Continuum Model of the Solvent Defined by the Bulk Dielectric Constant and Atomic Surface Tensions. *J. Phys. Chem. B* **2009**, *113*, 6378-6396.

[4] Luchini, G.; Alegre-Requena; IFunes; Rodríguez-Guerra, J.; Chen, J.; Paton, R. Bobbypaton /goodvibes: Goodvibes V3.0.0. Zenodo July 23, **2019**. https://doi.org/10.5281/zenodo.3346166.

[5] Svatunek, D.; Houk, K. N. autoDIAS: a python tool for an automated distortion/interaction activation strain analysis. *J. Comput. Chem.* **2019**, *40*, 2509-2515.

[6] Svatunek, D.; Denk, C.; Rosecker, V.; Sohr, B.; Hametner, C.; Allmaier, G.; Frohlich, J.; Mikula, H. Efficient low-cost preparation of trans-cyclooctenes using a simplified flow setup for photoisomerization. *Monatsh. Chem.* **2016**, *147*, 579-585.

[7] NBO 6.0., Glendening, E. D.; Badenhoop, J. K.; Reed, A. E.; Carpenter, J. E.; Bohmann, J. A.; Morales, C. M.; Landis, C. R.; Weinhold, F., Theoretical Chemistry Institute, University of Wisconsin, Madison, WI, 2013; http://nbo6.chem.wisc.edu/

[8] Eising, S.; Lelivelt, F.; Bonger, K. M. Vinylboronic Acids as Fast Reacting, Synthetically Accessible, and Stable Bioorthogonal Reactants in the Carboni-Lindsey Reaction. *Angew Chem Int Ed Engl* **2016**, *55*, 12243-7.

[9] Qu, Y.; Sauvage, F. X.; Clavier, G.; Miomandre, F.; Audebert, P. Metal-Free Synthetic Approach to 3-Monosubstituted Unsymmetrical 1,2,4,5-Tetrazines Useful for Bioorthogonal Reactions. *Angew Chem Int Ed Engl* **2018**, *57*, 12057-12061.

[10] Eising, S.; Xin, B. T.; Kleinpenning, F.; Heming, J. J. A.; Florea, B. I.; Overkleeft, H. S.; Bonger, K. M. Coordination-Assisted Bioorthogonal Chemistry: Orthogonal Tetrazine Ligation with Vinylboronic Acid and a Strained Alkene. *Chembiochem* **2018**, *19*, 1648-1652.

[11] Choi, A. W.; Tso, K. K.; Yim, V. M.; Liu, H. W.; Lo, K. K. Modification of 1,2,4,5-tetrazine with cationic rhenium(I) polypyridine units to afford phosphorogenic bioorthogonal probes with enhanced reaction kinetics. *Chem Commun (Camb)* **2015**, *51*, 3442-5.

[12] Xie, Y.; Fang, Y.; Huang, Z.; Tallon, A. M.; am Ende, C. W.; Fox, J. M. Divergent Synthesis of Monosubstituted and Unsymmetrical 3,6-Disubstituted Tetrazines from Carboxylic Ester Precursors. *Angewandte Chemie International Edition* **2020**, *59*, 16967-16973.

[13] Lambert, W. D.; Fang, Y.; Mahapatra, S.; Huang, Z.; am Ende, C. W.; Fox, J. M. Installation of Minimal Tetrazines through Silver-Mediated Liebeskind–Srogl Coupling with Arylboronic Acids. *Journal of the American Chemical Society* **2019**, *141*, 17068-17074.

[14] Stéen, E. J. L.; Jørgensen, J. T.; Denk, C.; Battisti, U. M.; Nørregaard, K.; Edem, P. E.; Bratteby, K.; Shalgunov, V.; Wilkovitsch, M.; Svatunek, D.; Poulie, C. B. M.; Hvass, L.; Simón, M.; Wanek, T.; Rossin, R.; Robillard, M.; Kristensen, J. L.; Mikula, H.; Kjaer, A.; Herth, M. M. Lipophilicity and Click Reactivity Determine the Performance of Bioorthogonal Tetrazine Tools in Pretargeted In Vivo Chemistry. *ACS Pharmacology & Translational Science* **2021**, *4*, 824-833.
